# The global, regional, and national disease burden of colorectal cancer attributable to low physical activity from 1990 to 2021: an analysis of the Global Burden of Disease Study 2021

**DOI:** 10.1007/s00384-025-04811-2

**Published:** 2025-01-18

**Authors:** Yanxue Lian, Alwaleed M. Alruwaili, Pincheng Luo

**Affiliations:** School of Medicine, University of Galway, Galway, Ireland

**Keywords:** Colorectal cancer, Physical activity, Global burden of disease, Cancer prevention

## Abstract

**Objectives:**

This study aims to estimate the spatiotemporal variation in the burden of colorectal cancer (CRC) attributable to low physical activity (LPA) at global, regional, and national levels from 1990 to 2021.

**Study design:**

Cross-sectional study.

**Methods:**

Annual data on deaths of CRC related to LPA, age-standardized mortality rate (ASMR), disability-adjusted life years (DALYs), and the age-standardized DALYs rate (ASDR) for 204 countries and territories from 1990 to 2021 was extracted from the Global Health Data Exchange website. They were retrieved by age (5-year age groups from 25 to 94 years, and 95+ years), gender (male and female), and Socio-demographic Index (SDI). The association between age-standardized rates and SDI values was assessed by Spearman’s correlation.

**Results:**

Between 1990 and 2021, there was nearly a twofold increase in DALYs and mortality globally for CRC related to LPA, despite decreases in ASMR and ASDR (EAPC: −0.82% and −0.83%, respectively). However, on a national scale, ASMR and ASDR increased in more than half of the world’s countries and territories. Moreover, a greater burden of CRC related to LPA was observed in older populations, females, and those residing in regions with an SDI near 0.77.

**Conclusion:**

These findings indicate the critical need to raise awareness about the preventive role of physical activity in CRC. Policymakers should prioritize developing and implementing strategies that ensure equitable access to sports resources, enabling more people to meet the World Health Organization guidelines.

**Supplementary Information:**

The online version contains supplementary material available at 10.1007/s00384-025-04811-2.

## Introduction

Colorectal cancer (CRC) ranked as the third most common cause of cancer-related deaths globally and was the second leading contributor to disability-adjusted life years (DALYs) among all cancers [1]. The age-standardized mortality rate (ASMR) and age-standardized DALYs rate (ASDR) of CRC decreased globally in the last few decades, mainly due to early diagnosis, cancer database development, enhanced technology, and standardization of early physician referrals [2]. However, its age-standardized incidence rate increased, probably owing to lifestyle changes, such as unhealthy dietary habits, substance addiction, sedentary behavior, and physical inactivity resulting from rapid economic progress and quick industrial advancement [2]. Being regularly active is one of the top recommendations to reduce cancer risk from the World Cancer Research Fund, which advises engaging in at least moderate physical activity and minimizing sedentary behavior [3]. However, given the increasing trend of insufficient physical activity globally, there is a need to enhance physical activity within health policies [4]. This enhancement will not only aid in the control of CRC but also decrease the risk associated with other health burdens [5].

As high-quality new evidence emerges on the link between physical activity and CRC risk, it is crucial to monitor and assess the disease burden of CRC attributable to low physical activity (LPA) [2]. Current research predominantly emphasizes the treatment and diagnosis of CRC [6–8]. Although the preventive effects of lifestyle modifications are acknowledged, the specific role of physical activity as a preventive measure has only recently gained recognition [9]. As a result, there is a notable lack of current and comprehensive data on the burden of LPA-related CRC across many countries and territories globally. To address this gap, this study aims to estimate the spatiotemporal variation in the burden of LPA-related CRC at global, regional, and national levels from 1990 to 2021. The findings will help inform the allocation of sports resources and the development of strategies to promote physical activity, as well as aid in the prevention and control of CRC.

## Method

### Study data

The Global Health Data Exchange website (https://vizhub.healthdata.org/gbd-results/) was utilized to collect annual data on CRC-related deaths, ASMR, DALYs, and ASDR for 204 countries and territories from 1990 to 2021. Data were gathered by age group (5-year intervals from 25 to 94 years, and 95+ years), gender (male and female), and region. All countries and territories were divided into five regions (low, low-middle, middle, high-middle, and high) based on the quintiles of the Socio-demographic Index (SDI), which represents the geometric mean of the total fertility rate under age 25, average years of education, and lag-distributed income per capita [10]. Additionally, countries and territories were assigned to 21 Global Burden of Disease Study (GBD) regions according to geographic classification [10]. Estimates were presented with 95% uncertainty intervals (UIs), derived from the 2.5th and 97.5th percentiles of 1000 ordered draws. Detailed methodologies for estimating CRC burden and attributable burden are found in the supplementary file. Moreover, given the usage of deidentified data in the GBD study, a waiver of informed consent has been approved by the University of Washington Institutional Review Board.

### Statistical analysis

The global, regional, and national burden of CRC attributable to LPA was evaluated using deaths, ASMR, DALYs, and ASDR. Trends in age-standardized rates (ASRs) from 1990 to 2021 were assessed using estimated annual percentage change (EAPC). A regression model, ln (ASR) = *α* + *βx* + *ε*, where *x* denotes the calendar year, was applied to the natural logarithm of ASR [11, 12]. EAPC was determined using the formula 100 × (exp(*β*) − 1), and the model also allowed for the calculation of its 95% confidence intervals (CIs) [11]. The ASR was considered stable if the 95% CIs included 0, decreasing if the EAPC, and its 95% CIs were < 0, and increasing if they were > 0. The association between ASR and SDI values was assessed by Spearman’s correlation. All statistical analyses were conducted in R software (version 4.0), with data visualization achieved through ggplot2, RcolorBrewer packages, and GraphPad Prism (version 9.5).

## Results

### Global trends

From 1990 to 2021, the global burden of CRC attributable to LPA surged significantly (Table 1). The number of deaths rose by 96%, from 36,497 to 71,476, while the burden measured in DALYs increased by approximately 83%, from 725,263 to 1,329,689. Despite this rise in absolute numbers, the ASMR declined from 1.09 per 100,000 individuals in 1990 to 0.87 per 100,000 individuals in 2021 (EAPC: −0.82%). Similarly, the ASDR decreased from 19.69 to 15.69 per 100,000 people during the past 31 years (EAPC: −0.83%).

**Table 1:** Deaths and DALYs of CRC attributable to LPA in 1990 and 2021 at global and SDI levels, with EAPC between 1990 and 2021

	1990				2021				EAPC	
	Death cases in 1990, no. (95% UI)	ASMR per 10^5^ in 1990 (95% UI)	DALYs in 1990, no. (95% UI)	ASDR per 10^5^ in 1990 (95% UI)	Death cases in 2021, no. (95% UI)	ASMR per 10^5^ in 2021 (95% UI)	DALYs in 2021, no. (95% UI)	ASDR per 10^5^ in 2021 (95% UI)	EAPC in ASMR (1990–2021)	EAPC in ASDR (1990–2021)
Global	36,497 (23,151 to 50,772)	1.09 (0.7 to 1.53)	725,263 (458,229 to 1,007,049)	19.69 (12.45 to 27.4)	71,476 (44,830 to 98,195)	0.87 (0.55 to 1.19)	1,329,689 (831,184 to 1,829,993)	15.69 (9.81 to 21.54)	−0.82% (−0.86 to −0.78)	−0.83% (−0.89 to −0.78)
Sex										
Male	12,883 (7912 to 17,918)	0.92 (0.57 to 1.30)	268,108 (165,239 to 370,163)	16.57 (10.18 to 22.94)	29,838 (18,372 to 42,034)	0.84 (0.52 to 1.19)	574,371 (348,379 to 800,745)	15.03 (9.21 to 20.93)	−0.32% (−0.36 to −0.29)	−0.37% (−0.43 to −0.32)
Female	23,614 (14,873 to 32,783)	1.21 (0.75 to 1.68)	457,156 (291,108 to 629,289)	22.15 (14.04 to 30.6)	41,638 (25,801 to 57,791)	0.89 (0.55 to 1.23)	755,318 (463,931 to 1,023,687)	16.29 (9.99 to 22.09)	−1.1% (−1.14 to −1.06)	−1.11% (−1.16 to −1.06)
SDI region										
High SDI	18,742 (11,794 to 26,442)	1.67 (1.05 to 2.36)	343,473 (214,268 to 477,490)	30.68 (19.12 to 42.73)	26,930 (16,365 to 37,115)	1.11 (0.68 to 1.52)	448,621 (277,707 to 612,777)	20.55 (12.8 to 27.97)	−1.41% (−1.46 to −1.35)	−1.4% (−1.47 to −1.33)
High-middle SDI	9734 (6159 to 13,346)	1.12 (0.72 to 1.55)	195,281 (122,259 to 267,597)	20.65 (12.98 to 28.34)	20,674 (12,797 to 28,471)	1.06 (0.66 to 1.45)	375,256 (232,754 to 530,140)	18.92 (11.73 to 26.64)	−0.24% (−0.29 to −0.18)	−0.36% (−0.39 to −0.32)
Middle SDI	5753 (3522 to 8143)	0.7 (0.43 to 0.99)	132,010 (80,679 to 185,252)	13.64 (8.4 to 19.31)	17,533 (10,992 to 24,247)	0.73 (0.46 to 0.99)	363,596 (227,978 to 507,256)	13.92 (8.76 to 19.39)	0% (−0.05 to 0.05)	−0.04% (−0.12 to 0.03)
Low-middle SDI	1717 (1064 to 2350)	0.34 (0.21 to 0.46)	41,292 (25,895 to 57,041)	7.04 (4.39 to 9.68)	5141 (3194 to 7093)	0.41 (0.25 to 0.57)	114,826 (70,846 to 158,795)	8.25 (5.12 to 11.41)	0.65% (0.6 to 0.71)	0.49% (0.44 to 0.53)
Low SDI	498 (290 to 722)	0.27 (0.16 to 0.39)	12,206 (6885 to 17,804)	5.64 (3.27 to 8.16)	1103 (690 to 1536)	0.27 (0.17 to 0.38)	25,667 (16,083 to 35,446)	5.38 (3.36 to 7.47)	0.04% (−0.06 to 0.15)	−0.21% (−0.32 to −0.11)
Age groups										
25–29 years	47 (25 to 85)	0.01 (0.01 to 0.02)	2994 (1573 to 5399)	0.68 (0.36 to 1.22)	49 (26 to 79)	0.01 (0 to 0.01)	3188 (1683 to 5094)	0.54 (0.29 to 0.87)	−0.69% (−0.97 to −0.4)	−0.65% (−0.93 to −0.36)
30–34 years	93 (51 to 162)	0.02 (0.01 to 0.04)	5473 (2996 to 9483)	1.42 (0.78 to 2.46)	123 (70 to 207)	0.02 (0.01 to 0.03)	7344 (4197 to 12,264)	1.21 (0.69 to 2.03)	−0.61% (−0.86 to −0.36)	−0.57% (−0.82 to −0.32)
35–39 years	176 (94 to 295)	0.05 (0.03 to 0.08)	9480 (5067 to 15,870)	2.69 (1.44 to 4.51)	221 (123 to 345)	0.04 (0.02 to 0.06)	12,042 (6709 to 18,819)	2.15 (1.2 to 3.36)	−0.74% (−1.01 to −0.47)	−0.71% (−0.97 to −0.43)
40–44 years	298 (173 to 450)	0.1 (0.06 to 0.16)	14,620 (8526 to 22,040)	5.1 (2.98 to 7.69)	412 (240 to 602)	0.08 (0.05 to 0.12)	20,400 (11,833 to 29,904)	4.08 (2.37 to 5.98)	−0.78% (−1.1 to −0.46)	−0.74% (−1.06 to −0.43)
45–49 years	529 (326 to 779)	0.23 (0.14 to 0.34)	23,262 (14,400 to 34,151)	10.02 (6.2 to 14.71)	851 (510 to 1200)	0.18 (0.11 to 0.25)	37,850 (22,689 to 53,597)	7.99 (4.79 to 11.32)	−0.85% (−1.09 to −0.6)	−0.81% (−1.06 to −0.56)
50–54 years	1001 (583 to 1473)	0.47 (0.27 to 0.69)	39,198 (22,819 to 57,698)	18.44 (10.73 to 27.14)	1652 (953 to 2340)	0.37 (0.21 to 0.53)	65,568 (37,711 to 93,085)	14.74 (8.48 to 20.92)	−0.92% (−1.1 to −0.73)	−0.87% (−1.06 to −0.69)
55–59 years	1794 (1027 to 2600)	0.97 (0.55 to 1.4)	61,720 (35,489 to 89,657)	33.33 (19.16 to 48.41)	2997 (1768 to 4387)	0.76 (0.45 to 1.11)	104,678 (61,844 to 153,287)	26.45 (15.63 to 38.74)	−0.92% (−1.05 to −0.8)	−0.87% (−1 to −0.75)
60–64 years	3032 (1805 to 4323)	1.89 (1.12 to 2.69)	90,097 (53,989 to 128,316)	56.1 (33.62 to 79.89)	4626 (2707 to 6559)	1.45 (0.85 to 2.05)	139,532 (82,511 to 199,466)	43.6 (25.78 to 62.32)	−0.97% (−1.08 to −0.85)	−0.91% (−1.03 to −0.8)
65–69 years	4232 (2583 to 5878)	3.42 (2.09 to 4.76)	106,376 (65,065 to 148,308)	86.06 (52.64 to 119.98)	7238 (4318 to 10,235)	2.62 (1.57 to 3.71)	184,702 (111,647 to 259,669)	66.96 (40.47 to 94.14)	−0.99% (−1.08 to −0.91)	−0.93% (−1.02 to −0.85)
70–74 years	4998 (3010 to 6867)	5.9 (3.56 to 8.11)	103,646 (62,734 to 142,506)	122.42 (74.1 to 168.32)	9895 (6026 to 13,999)	4.81 (2.93 to 6.8)	208,653 (127,309 to 295,254)	101.37 (61.85 to 143.44)	−0.96% (−1.04 to −0.87)	−0.91% (−0.99 to −0.82)
75–79 years	6810 (4159 to 9433)	11.06 (6.76 to 15.32)	113,002 (68,925 to 156,947)	183.58 (111.97 to 254.97)	10,854 (6565 to 15,306)	8.23 (4.98 to 11.61)	183,225 (110,716 to 256,911)	138.93 (83.95 to 194.8)	−0.96% (−1.02 to −0.9)	−0.92% (−0.98 to −0.86)
80–84 years	7101 (4425 to 9916)	20.07 (12.51 to 28.03)	92,505 (58,030 to 130,223)	261.49 (164.04 to 368.11)	13,513 (8352 to 18,568)	15.43 (9.54 to 21.2)	177,680 (110,486 to 243,419)	202.87 (126.15 to 277.93)	−0.87% (−0.93 to −0.8)	−0.84% (−0.9 to −0.77)
85–89 years	4243 (2563 to 6018)	28.08 (16.96 to 39.83)	43,994 (26,835 to 62,353)	291.14 (177.59 to 412.63)	10,554 (6286 to 14,621)	23.08 (13.75 to 31.98)	110,267 (66,077 to 151,990)	241.17 (144.52 to 332.42)	−0.65% (−0.72 to −0.58)	−0.62% (−0.69 to −0.55)
90–94 years	1692 (1011 to 2416)	39.49 (23.6 to 56.39)	15,118 (9061 to 21,625)	352.79 (211.45 to 504.65)	6129 (3682 to 8554)	34.26 (20.58 to 47.81)	55,046 (33,123 to 76,727)	307.7 (185.16 to 428.9)	−0.51% (−0.61 to −0.41)	−0.49% (−0.58 to −0.39)
95+ years	451 (259 to 655)	44.25 (25.45 to 64.32)	3778 (2179 to 5475)	371.13 (214.02 to 537.72)	2361 (1380 to 3378)	43.33 (25.32 to 61.97)	19,515 (11,550 to 28,003)	358.05 (211.91 to 513.78)	−0.23% (−0.34 to −0.12)	−0.29% (−0.4 to −0.17)

*ASDR* age-standardized DALYs rate, *ASMR* age-standardized mortality rate, *DALYs* disability-adjusted life years, *EAPC* estimated annual percentage change, *SDI* Socio-demographic Index, *UIs* uncertainty intervals

### Global trends by sex

In both 1990 and 2021, the number of deaths among females was nearly double that of males (Table 1). While the ASMR decreased in both genders, the decline was more pronounced in females than in males (EAPC: −1.1% vs. −0.32%). Despite this faster decline, women consistently had a higher ASMR than men. This gender disparity persisted not only globally but also in all regions besides the high-middle and middle SDI regions (see Supplementary Table 1 and Supplementary Figure 1). Similarly, the DALYs for women were approximately 1.5 times higher than those for men in both 1990 and 2021. The decline in the ASDR among females was also steeper compared to males (EAPC: −1.11% vs. −0.37%) (see Supplementary Figure 2). As with mortality rates, women had a higher ASDR than men globally, with this trend persisting in all SDI regions except the high-middle SDI region (see Supplementary Table 2 and Supplementary Figure 1).

### Global trends by SDI index

From 1990 to 2021, mortality and DALYs increased across all SDI subgroups, with deaths rising most sharply in the middle SDI region and DALYs in the low-middle SDI region. The high SDI region consistently exhibited the highest burden of mortality and DALYs (Table 1). Moreover, although regions with higher SDIs had higher ASMR and ASDR, the greatest decline occurred in the high SDI region, while the low-middle SDI region saw the largest increase (see supplementary figure 1 and supplementary figure 2).

**Fig. 1:**
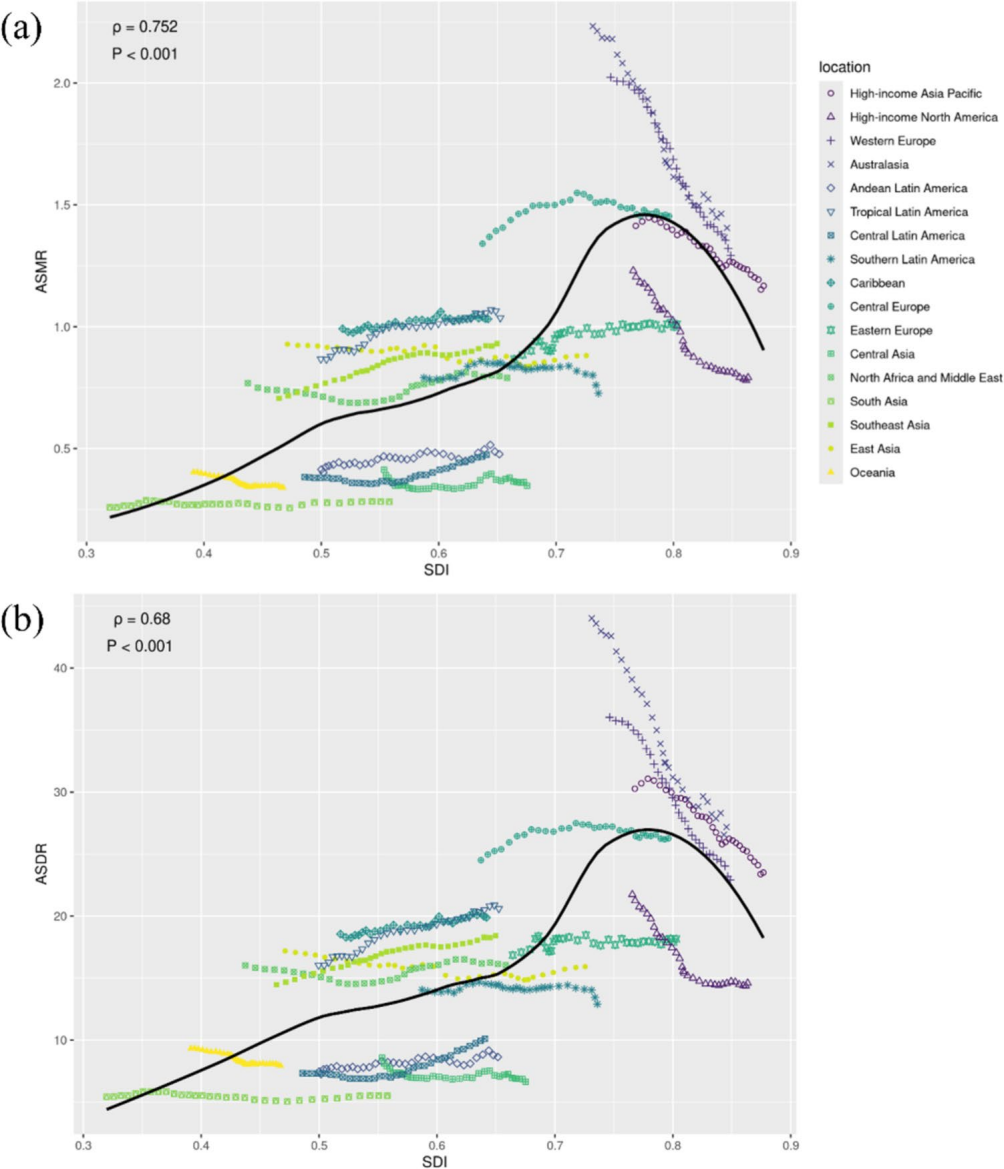
The relationship between burden of CRC attributable to LPA and the SDI in 2021 by GBD regions. The relationship between **a** ASMR and **b** ASDR of CRC attributable to LPA and the SDI in 2021 by GBD regions. Abbreviations: ASMR, age-standardized mortality rate; ASDR, age-standardized DALYs rate; SDI, Socio-demographic Index

Generally, among these 31 years, the evolution of ASMR of LPA-related CRC in relation to SDI across different GBD regions progressed through distinct phases. Initially, there was a period of linear increase as SDI approached approximately 0.66. Following this, ASMR experienced logarithmic growth until SDI reached about 0.77. Subsequently, there was a notable rapid decline in ASMR, mirroring the symmetrical pattern observed during the logarithmic growth phase at an SDI of 0.77. A similar pattern was found between ASDR and SDI (Figure 1).

### Global trends by age subgroups

Globally, the number of deaths from CRC attributable to LPA increased in every age group from 1990 to 2021 (Table 1). Especially, it increased by 4–5 times in people aged ≥ 90 years while increased by 1.5–2 times in those aged 75 to 89 years. Although the mortality rates per 100,000 population increased with age (see Supplementary figure 3), the age-specific mortality rate decreased in all age subgroups with the greatest decrease in the population aged 65–69 years (EAPC: −0.99%). Moreover, from the age group 30–34 to 65–69 years, the rate of decline in mortality increased progressively with age, but from the age group 65–69 to 95+ years, the rate of decline in mortality slowed down, forming an inverted V shape. Similarly, age-specific DALY rates increased with age, with the most notable decline occurring among those aged 65–69 years between 1990 and 2021 (EAPC: −0.93%). Additionally, both age-specific rates of mortality and DALYs decreased in all age subgroups among females, but this was not consistent among males (see supplementary figure 4).

### Regional trends

At the regional level according to the GBD study, deaths attributed to CRC associated with LPA were mainly concentrated in Western Europe, comprising 33.97% of global deaths in 1990 (see supplementary table 3). By 2021, East Asia and Western Europe emerged as the top two regions with the highest number of deaths, accounting for 24.57% and 20.61% of global CRC deaths related to LPA, respectively. From 1990 to 2021, a decline in ASMR was observed in six regions, with the EAPC ranging from −1.63 to −0.27%, while a relatively stable ASMR was noticed in two regions. In contrast, ASMRs in other GBD regions showed an upward trend, with the most rapid increase noted in Southeast Asia (EAPC: 0.81%).

In 2021, there were four regions surpassing 100 thousand DALYs. However, in 1990, only East Asia and Western Europe exceeded this threshold. Notably, although Australasia consistently had the highest ASDR, at 44.01 per 100,000 in 1990 and 27.18 per 100,000 in 2021, it experienced the most significant decline over these 31 years (EAPC: −1.69%). This trend persisted after sex stratification, with EAPCs in ASDR of −1.68% for males and −1.71% for females. Additionally, substantial declines in ASDRs were also seen in Western Europe and High-income North America, with EAPCs of −1.53% and −1.41%, respectively. On the other hand, upward trends were observed in ten regions, with the most rapid increases in Central Latin America (EAPC: 1.11%) (see supplementary table 3 and supplementary figure 5).

### National trends

**Fig. 2:**
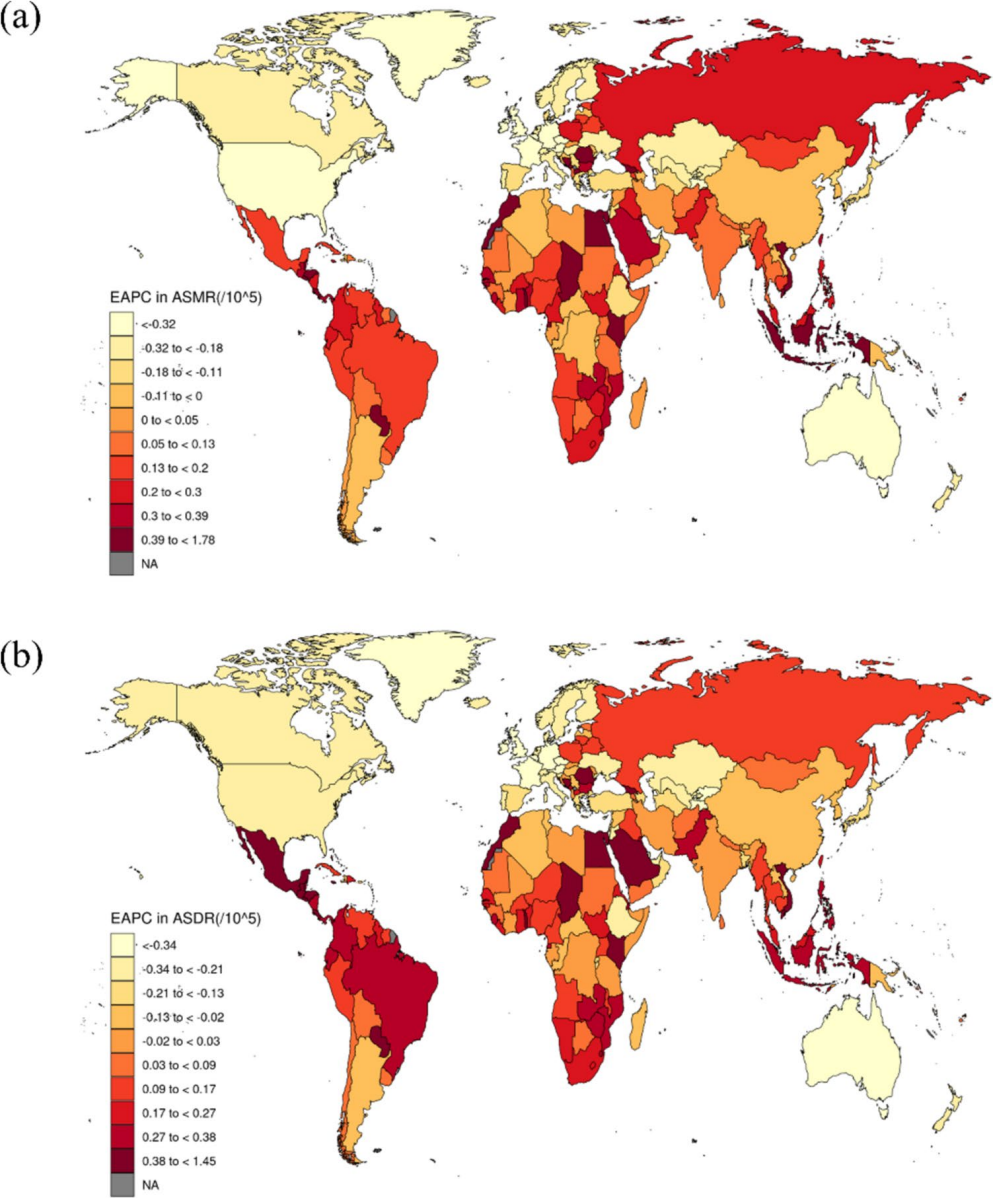
Map of EAPC in ASR of CRC attributable to LPA from 1990 to 2021. **a** Showing EAPC in ASMR of CRC attributable to LPA from 1990 to 2021 and **b** showing EAPC in ASDR of CRC attributable to LPA from 1990 to 2021. Abbreviations: EAPC, estimated annual percentage change; ASMR, age-standardized mortality rate; ASDR, age-standardized DALYs rate

At the national level, China recorded the highest number of CRC deaths attributable to LPA in both 1990 (5735) and 2021 (16698). The UK had the highest ASMR in 1990 (3.37 per 100,000), with Barbados showing the highest ASMR in 2021 (2.41 per 100,000). In 1990, five countries had the lowest ASMR of 0.09 per 100,000. The United Republic of Tanzania maintained the lowest ASMR in 2021, at 0.09 per 100,000. Between 1990 and 2021, ASMRs increased in 114 countries and territories, with Cabo Verde showing the fastest rise (EAPC: 2.97%). ASMRs remained stable in ten countries and territories, while they declined in 79 others (Figure 2 and supplementary table 4).

In terms of DALYs, China and Japan consistently ranked as the top two countries. From 1990 to 2021, the DALYs in both countries increased, by 1.5% and 0.70%, respectively. Australia had the highest ASDR in 1990, at 44.71 per 100,000, while Barbados held this position in 2021, with the ASDR of 46.69 per 100,000. In 1990, Mozambique had the lowest ASDR, at 1.61 per 100,000, but by 2021, the United Republic of Tanzania had replaced Mozambique in this position with the ASDR of 1.71 per 100,000. Over the 31-year period, 78 countries and territories saw a reduction in ASDR, with Austria showing the largest decline (EAPC: −2.75). Additionally, 21 countries and territories maintained a stable ASDR. In contrast, 104 countries and territories experienced an increase in ASDR, with Lesotho recording the most significant rise (EAPC: 2.68%) (Figure 2 and supplementary table 4).

## Discussion

This study provides global, regional, and national estimates of spatiotemporal trends in mortality and DALYs attributed to CRC associated with LPA. Between 1990 and 2021, there was nearly a twofold increase in DALYs and mortality globally for CRC related to LPA, despite decreases in ASMR and ASDR. However, on a national scale, ASMR and ASDR increased in more than half of the world’s countries and territories. Moreover, a greater burden of CRC related to LPA was observed in females and older age subgroups. Initially increasing, the burden of LPA-related CRC subsequently decreased, reaching its peak burden at an SDI near 0.77. These findings enhance the current understanding of the rising global burden of LPA prevalence by examining trends in CRC attributed to LPA. Additionally, this highlights the urgency of promoting physical activity as a key preventive measure, with effective strategies prioritizing populations at higher risk. Efforts should focus on promoting physical activity across populations, enhancing cancer surveillance and early detection efforts, and addressing disparities in healthcare access and outcomes to effectively reduce the impact of CRC attributable to LPA globally.

Extensive research has shown that there is an inverse relationship between physical activity and CRC risk. A prospective study based on the US and European cohorts revealed that individuals with high levels of physical activity had a hazard ratio (HR) of 0.84 for colon cancer risk compared to those with low levels, a trend that persisted even after adjusting for body mass index and smoking status [13]. A similar result was reported with a HR of 0.85 in a study conducted in Singapore Chinese [14]. Furthermore, a study utilizing 24-h accelerometry time series from the UK Biobank examined the influence of circadian physical activity patterns on CRC risk. Both a continuous day-long activity pattern (HR = 0.94) and an early-plus late-day activity pattern (HR = 0.89) were associated with a reduced risk of CRC [15]. In addition to its preventative effects, maintaining a high level of physical activity is beneficial for CRC survivors. Being physically active is not only associated with a reduced recurrence of polyps and advanced adenomas [16] but also positively related to postoperative survival rates [17]. Hong and Park (2021) recommended that engaging in physical activity levels ranging from 17.5 to 35 MET hours per week was highly advisable for individuals diagnosed with CRC, as it could reduce mortality rates by about 30 to 40% [18]. Those with limited physical abilities should aim to sustain at least a minimum of 3.5 MET hours per week [18].

### Age differences in burden of LPA-related CRC

From 1990 to 2021, the mortality rate associated with CRC due to LPA decreased, and the life expectancy of affected individuals increased across all age subgroups. These trends may be attributed to recent advancements in developmental objectives focused on enhancing the accessibility and scope of healthcare services, alongside improvements in economic growth, poverty reduction, and social welfare initiatives [19]. Despite the overall decrease in CRC mortality rates, age-specific mortality and DALYs increased with age, highlighting the disproportionately greater burden of LPA-related CRC among the elderly compared to younger populations.

As individuals age, biological systems undergo substantial changes, including a decline in immune function and diminished cellular repair mechanisms, which heighten cancer susceptibility [20]. Chronic conditions such as heart disease, diabetes, and respiratory disorders are more prevalent in older adults, limiting their capacity for aggressive cancer therapies and reducing stamina and physical resilience [21]. Mobility challenges stemming from arthritis, osteoporosis, or general muscle weakness further hinder physical activity [22], compounded by fears of falls or injuries [23]. Environmental factors also exacerbate these barriers; many elderly individuals live in areas lacking safe walking paths, accessible parks, or adequate transportation options, limiting opportunities for physical or community-based activities [24]. Additionally, perceptions of reduced physical capability and fatigue lower self-efficacy, discouraging older adults from initiating or maintaining regular physical activity [25].

Together, these physiological, behavioral, and environmental challenges, coupled with the global trend of population aging, help explain the disproportionate burden of LPA-related CRC among the elderly. Addressing this requires targeted strategies, including tailored exercise programs and balance training to reduce fall risks, alongside urban planning to ensure access to parks, walking paths, and transportation. Peer-led exercise groups and family-based activities can enhance social support and integration of physical activity into daily routines. Successful initiatives like Japan’s Fujisawa +10 Exercise Program, which encourages older adults to add 10 min of physical activity to their daily routines through simple and accessible exercises, demonstrate the effectiveness of community-focused approaches in promoting active lifestyles [26]. Routine CRC screening is also essential for early detection and intervention, which can mitigate disease burden, especially in the elderly population. Finally, healthcare providers should actively encourage exercise and screening, supported by policies that reduce barriers to activity and promote healthy aging.

### Sex differences in burden of LPA-related CRC

While CRC incidence is higher among males [27], females bore a greater burden of CRC linked to LPA globally and regionally from 1990 to 2021. This suggests a significant disparity in physical activity levels between the sexes, with females being less active than males. Previous studies consistently report that females exhibit 6–10% lower physical activity levels than males, regardless of the measurement instruments, protocols, or definitions used [28–30]. This difference persists throughout the lifespan, spanning from prenatal stages [31], through infancy [32–34], childhood [35], adolescence [30], adulthood [31], to old age [36]. The sex disparity of physical activity could be attributed to several factors.

Sociocultural and environmental factors significantly contribute to the sex disparity in physical activity, as family dynamics, safety concerns, cultural norms, and media representation often limit women’s participation. In many cultures, higher employment rates lead to increased occupation- and transportation-related physical activity in men [37] and women’s time constraints as primary caregivers [38]. Family emphasis on academic performance rather than sports further discourages physical activity. Safety concerns, such as the fear of harassment or violence in public spaces, restrict women’s engagement in outdoor activities, pushing them toward more sedentary behaviors [39]. Cultural and religious practices, including restrictive dress codes and discouragement of mixed-gender sports, also limit women’s opportunities for physical activity [40]. Additionally, the media often portrays sports as a male-dominated domain, with limited representation of female athletes, reducing the visibility of role models and reinforcing the perception that physical activity is not for women [41]. These sociocultural barriers collectively contribute to lower physical activity levels among women.

Biological factors also play a crucial role in shaping the sex disparity in physical activity by affecting women’s physical abilities, overall health, energy levels, and mental well-being. Hormonal fluctuations across various life stages—such as puberty, menstruation, pregnancy, and menopause—can impact energy levels, mood, and motivation, often reducing the likelihood of consistent physical activity [42]. Moreover, women generally have lower vital capacity, muscle mass, and upper-body strength compared to men, which may influence their preference for less physically demanding activities [42]. Emerging evidence also suggests that biological and physiological factors may shape gender-specific behaviors, including differences in preferences and interest in physical activity [42]. Additionally, psychological factors such as body image and self-esteem intersect with these biological influences [42]. Societal pressures surrounding physical appearance can undermine women’s confidence and self-efficacy, further limiting their participation in physical activity and perpetuating the disparity between men and women.

To reduce the sex disparity in physical activity and lower the burden of LPA-related CRC in women, targeted interventions are essential. Community-based programs offering women-only fitness classes in culturally appropriate settings can create safe spaces for participation. Time-efficient routines like home-based high-intensity interval training can help women manage physical activity alongside caregiving and work. Family engagement in joint physical activities, media campaigns to reshape body image perceptions, and policies to improve outdoor safety and media representation of female athletes can further support participation. Life-stage-specific interventions, such as prenatal yoga, postpartum fitness, and strength training during menopause, can address hormonal and physiological challenges, promoting consistent physical activity and reducing CRC risk.

### Socio-demographic differences in burden of LPA-related CRC

Significant disparities in the burden of CRC attributable to LPA exist across countries and regions, closely tied to levels of socio-demographic development. Over the past three decades, the burden initially increased with rising socioeconomic development, followed by a gradual decline in highly developed regions. This trend reflects a complex interplay of socioeconomic, healthcare, lifestyle, and environmental factors. In countries with lower levels of socio-demographic development, the initial rise in LPA-related CRC burden is primarily driven by lifestyle changes associated with economic development and urbanization. Rapid industrialization often shifts populations from physically demanding occupations to more sedentary work environments [43, 44], accompanied by increased reliance on motorized transport and reduced opportunities for recreational physical activity [45]. These changes, combined with dietary transitions toward high-calorie, low-fiber diets [46], significantly elevate CRC risk. Healthcare access is another critical factor, as limited screening services and financial constraints delay diagnosis [47], leading to higher CRC mortality rates. Furthermore, urbanization in these regions often lacks supportive infrastructure for physical activity, such as green spaces, safe pedestrian areas, and recreational facilities, further discouraging active lifestyles [48]. The CRC burden in some of these regions may also be underestimated due to the late establishment of cancer surveillance systems [49, 50].

In contrast, regions with higher levels of socio-demographic development have experienced a decline in the burden of CRC related to LPA. This trend can be attributed to increased public awareness of the health benefits of physical activity, widespread health promotion campaigns, and the establishment of a wellness culture [51]. Economic stability in these regions also allows individuals to engage in structured physical activity, such as gym memberships, sports participation, and active commuting [45], which collectively reduce CRC risk. Advanced healthcare infrastructure in these regions facilitates early detection and management of CRC through regular screening programs, such as colonoscopies and fecal occult blood tests [52]. Early diagnosis and timely intervention reduce CRC incidence and mortality, even in populations with suboptimal physical activity levels [52]. Investments in urban planning, such as the development of parks, bike lanes, and public transportation systems, also encourage active living [45].

Addressing CRC disparities requires regional interventions tailored to socioeconomic contexts. In less developed regions, expanding access to physical activity through community-based programs, such as free exercise classes, school-based initiatives, and workplace wellness schemes, is essential [45]. Strengthening healthcare infrastructure with mobile screening units, telemedicine, and subsidized screenings can improve early CRC detection. Urban planning should prioritize pedestrian-friendly spaces, cycling paths, and parks to promote active living and counter sedentary lifestyles [45]. Successful examples like Colombia’s Ciclovía program, which transforms city streets into car-free zones for walking, cycling, and community fitness on specific days each week, demonstrate the potential of inclusive urban interventions in promoting physical activity [53]. Mobile health apps and wearable devices offer cost-effective tools to encourage physical activity, while culturally tailored campaigns can enhance community engagement [54]. Finally, robust cancer surveillance systems are vital for monitoring CRC burden and guiding public health strategies.

### Strength and limitations

Epidemiological and clinical research has significantly advanced the understanding of physical activity’s protective effects, contributing to a decline in the CRC burden related to LPA over the past 31 years. This analysis evaluates intervention effectiveness and reveals disparities across SDI regions, genders, and age groups, emphasizing the need for better physical activity surveillance and public health strategies, particularly for women, older adults, and high-middle SDI countries. Despite World Health Organization (WHO) recommendations for adults to engage in at least 150–300 min of moderate or 75–150 min of vigorous activity weekly, or some equivalent combination of both [55], 27.5% of the population falls short [4], indicating a need for continued efforts to boost physical activity.

However, this study has limitations. First, the physical activity data in the GBD study were collected using standardized questionnaires. Despite their widespread use, these self-reported data are subject to biases stemming from social desirability, recall errors, and variations in health literacy and awareness, which may affect the accuracy of reported physical activity levels. This reliance on self-reports introduces uncertainty in the estimation of MET minutes per week. Future iterations of the GBD dataset could be enhanced by incorporating objective measures, such as accelerometer-based data, as they become more accessible, to improve the precision and reliability of physical activity estimates. Second, the assessment of CRC burden in low- and middle-income countries (LMICs) is influenced by several factors, including underreporting, data limitations, socioeconomic disparities, and unequal access to healthcare and research infrastructure. These factors contribute to inaccuracies in CRC burden estimates and highlight the need for more comprehensive data collection systems tailored to the specific challenges faced by LMICs. Addressing these gaps is essential for developing effective public health policies and targeted interventions in these regions. Third, the cross-sectional design of this study limits the ability to infer causality between LPA and CRC outcomes. While the findings illustrate trends in LPA-related CRC mortality and disease burden, they cannot establish direct causal links between individual physical activity levels and health outcomes. Additionally, the data are subject to a time lag inherent in the reporting of disease burden. Future research employing longitudinal and multilevel approaches is necessary to clarify the causal pathways between physical activity, social determinants, and CRC risk, providing a stronger evidence base for preventive strategies.

## Conclusion

In conclusion, mortality and DALYs for LPA-related CRC increased from 1990 to 2021, particularly among older populations, females, and those residing in regions with an SDI near 0.77. These findings indicate the critical need to raise awareness about the preventive role of physical activity in CRC. Policymakers should prioritize developing and implementing strategies that ensure equitable access to sports resources, enabling more people to meet the WHO guidelines of 150–300 min of moderate-intensity or 75–150 min of vigorous-intensity physical activity, or an equivalent combination, per week for adults.

## Supplementary Information

Below is the link to the electronic supplementary material.

Supplementary file1 (DOCX 1694 KB)

## Data Availability

The data used for analyses are publicly available at https://ghdx.healthdata.org/gbd-results-tool. All data will be available on request to the corresponding author.

## References

[1] Vos T, Lim SS, Abbafati C et al (2020) Global burden of 369 diseases and injuries in 204 countries and territories, 1990–2019: a systematic analysis for the Global Burden of Disease Study 2019. Lancet 396:1204–1222 33069326 10.1016/S0140-6736(20)30925-9 PMC7567026

[2] Sharma R, Abbasi-Kangevari M, Abd-Rabu R et al (2022) Global, regional, and national burden of colorectal cancer and its risk factors, 1990–2019: a systematic analysis for the Global Burden of Disease Study 2019. Lancet Gastroenterol Hepatol 7:627–647 35397795 10.1016/S2468-1253(22)00044-9 PMC9192760

[3] World Cancer Research Fund/American Institute for Cancer Research. Diet, nutrition, physical activity and cancer: a global perspective. Continuous Update Project Expert Report 2018., https://www.wcrf.org/diet-activity-and-cancer/cancer-prevention-recommendations/be-physically-active/ (2018, Accessed 6 August 2024).

[4] Guthold R, Stevens GA, Riley LM et al (2018) Worldwide trends in insufficient physical activity from 2001 to 2016: a pooled analysis of 358 population-based surveys with 1·9 million participants. Lancet Glob Health 6:e1077–e1086 30193830 10.1016/S2214-109X(18)30357-7

[5] Kerr J, Anderson C, Lippman SM (2017) Physical activity, sedentary behaviour, diet, and cancer: an update and emerging new evidence. Lancet Oncol 18:e457–e471 28759385 10.1016/S1470-2045(17)30411-4 PMC10441558

[6] Mayer RJ (2004) Two steps forward in the treatment of colorectal cancer. N Engl J Med 350:2406–2408 15175443 10.1056/NEJMe048098

[7] Wolpin BM, Mayer RJ (2008) Systemic treatment of colorectal cancer. Gastroenterology 134:1296-1310.e1 18471507 10.1053/j.gastro.2008.02.098 PMC2528832

[8] Biller LH, Schrag D (2021) Diagnosis and treatment of metastatic colorectal cancer. JAMA 325:669 33591350 10.1001/jama.2021.0106

[9] Chan AT, Giovannucci EL (2010) Primary prevention of colorectal cancer. Gastroenterology 138:2029-2043.e10 20420944 10.1053/j.gastro.2010.01.057 PMC2947820

[10] Wang H, Abbas KM, Abbasifard M et al (2020) Global age-sex-specific fertility, mortality, healthy life expectancy (HALE), and population estimates in 204 countries and territories, 1950–2019: a comprehensive demographic analysis for the Global Burden of Disease Study 2019. Lancet 396:1160–1203 33069325 10.1016/S0140-6736(20)30977-6 PMC7566045

[11] Yang X, Fang Y, Chen H et al (2021) Global, regional and national burden of anxiety disorders from 1990 to 2019: results from the Global Burden of Disease Study 2019. Epidemiol Psychiatr Sci 30:e36 33955350 10.1017/S2045796021000275 PMC8157816

[12] Hankey BF, Ries LA, Kosary CL et al (2000) Partitioning linear trends in age-adjusted rates. Cancer Causes Control 11:31–35 10680727 10.1023/a:1008953201688

[13] Moore SC, Lee I-M, Weiderpass E et al (2016) Association of leisure-time physical activity with risk of 26 types of cancer in 1.44 million adults. JAMA Intern Med 176:816 27183032 10.1001/jamainternmed.2016.1548 PMC5812009

[14] Eaglehouse YL, Koh W-P, Wang R et al (2017) Physical activity, sedentary time, and risk of colorectal cancer: the Singapore Chinese Health Study. Eur J Cancer Prev 26:469–475 28542077 10.1097/CEJ.0000000000000369 PMC5620106

[15] Stein MJ, Baurecht H, Bohmann P et al (2024) Diurnal timing of physical activity and risk of colorectal cancer in the UK Biobank. BMC Med 22:399 39289682 10.1186/s12916-024-03632-4 PMC11409794

[16] Park J, Kim JH, Lee HJ et al (2017) The effects of physical activity and body fat mass on colorectal polyp recurrence in patients with previous colorectal cancer. Cancer Prev Res 10:478–484 10.1158/1940-6207.CAPR-17-0065 28584169

[17] Hardikar S, Newcomb PA, Campbell PT et al (2015) Prediagnostic physical activity and colorectal cancer survival: overall and stratified by tumor characteristics. Cancer Epidemiol Biomark Prev 24:1130–1137 10.1158/1055-9965.EPI-15-0039 PMC4491038 25976417

[18] Oremus M, Dayes I, Walker K, Raina P (2012) Systematic review: conservative treatments for secondary lymphedema. BMC Cancer 12:6. 10.1186/1471-2407-12-6 10.1186/1471-2407-12-6 PMC3320521 22216837

[19] Marmot M (2015) The health gap: the challenge of an unequal world. Lancet 386:2442–2444 26364261 10.1016/S0140-6736(15)00150-6

[20] Montecino-Rodriguez E, Berent-Maoz B, Dorshkind K (2013) Causes, consequences, and reversal of immune system aging. J Clin Investig 123:958–965 23454758 10.1172/JCI64096 PMC3582124

[21] Extermann M (2007) Interaction between comorbidity and cancer. Cancer Control 14:13–22 17242667 10.1177/107327480701400103

[22] Brahms CM, Hortobágyi T, Kressig RW et al (2021) The interaction between mobility status and exercise specificity in older adults. Exerc Sport Sci Rev 49:15–22 33044331 10.1249/JES.0000000000000237

[23] Deshpande N, Metter JE, Lauretani F et al (2009) Interpreting fear of falling in the elderly: what do we need to consider? J Geriatric Phys Therapy 32:91–96 10.1519/00139143-200932030-00002 PMC2954585 20128332

[24] Maresova P, Krejcar O, Maskuriy R et al (2023) Challenges and opportunity in mobility among older adults – key determinant identification. BMC Geriatr 23:447 37474928 10.1186/s12877-023-04106-7 PMC10360303

[25] McAuley E, Szabo A, Gothe N et al (2011) Self-efficacy: implications for physical activity, function, and functional limitations in older adults. Am J Lifestyle Med 5:361–369 10.1177/1559827610392704 PMC3864698 24353482

[26] Saito Y, Tanaka A, Tajima T et al (2021) A community-wide intervention to promote physical activity: a five-year quasi-experimental study. Prev Med (Baltim) 150:106708 10.1016/j.ypmed.2021.106708 34197869

[27] Gangireddy VGR, Talla S (2018) Gender disparities in the incidence of colorectal cancer in the era of screening colonoscopy: 176. Official journal of the American College of Gastroenterology | ACG; 113, https://journals.lww.com/ajg/fulltext/2018/10001/gender_disparities_in_the_incidence_of_colorectal.176.aspx

[28] Hallal PC, Andersen LB, Bull FC et al (2012) Global physical activity levels: surveillance progress, pitfalls, and prospects. Lancet 380:247–257 22818937 10.1016/S0140-6736(12)60646-1

[29] Trost SG, Owen N, Bauman AE et al (2002) Correlates of adults’ participation in physical activity: review and update. Med Sci Sports Exerc 34:1996–2001 12471307 10.1097/00005768-200212000-00020

[30] Trost SG, Pate RR, Sallis JF et al (2002) Age and gender differences in objectively measured physical activity in youth. Med Sci Sports Exerc 34:350–355 11828247 10.1097/00005768-200202000-00025

[31] Almli CR, Ball RH, Wheeler ME (2001) Human fetal and neonatal movement patterns: gender differences and fetal-to-neonatal continuity. Dev Psychobiol 38:252–273 11319731 10.1002/dev.1019

[32] Campbell DW, Eaton WO (1999) Sex differences in the activity level of infants. Infant Child Dev 8:1–17

[33] Goldberg S, Lewis M (1969) Play behavior in the year-old infant: early sex differences. Child Dev 40:21 5787704

[34] Hutt C (1972) Sex differences in human development. Hum Dev 15:153–170 5042947 10.1159/000271239

[35] Pate RR, McIver K, Dowda M et al (2008) Directly observed physical activity levels in preschool children. J Sch Health 78:438–444 18651931 10.1111/j.1746-1561.2008.00327.x

[36] Lee Y-S (2005) Gender differences in physical activity and walking among older adults. J Women Aging 17:55–70 15914419 10.1300/J074v17n01_05

[37] Yang L, Hu L, Hipp JA et al (1978) Cross-sectional associations of active transport, employment status and objectively measured physical activity: analyses from the National Health and Nutrition Examination Survey. J Epidemiol Community Health 2018(72):764–769 10.1136/jech-2017-210265 PMC6086741 29730607

[38] Caperchione CM, Chau S, Walker GJ et al (2015) Gender-associated perceptions of barriers and motivators to physical activity participation in South Asian Punjabis living in Western Canada. J Phys Act Health 12:686–693 25105245 10.1123/jpah.2013-0208

[39] Eyler AA, Vest JR (2002) Environmental and policy factors related to physical activity in rural white women. Women Health 36:111–121 12487144

[40] Raza M, Ya Ling H, Hamdani SMZH et al (2022) Socio-cultural interest and motivational barriers for female sports participation in Pakistan: a comparative study of universities and colleges. Sustain Bus Soc Emerg Econ 4:547–560. 10.26710/sbsee.v4i2.2393

[41] Rasmussen K, Dufur MJ, Cope MR et al (2021) Gender marginalization in sports participation through advertising: the case of Nike. Int J Environ Res Public Health 18:7759 34360052 10.3390/ijerph18157759 PMC8345737

[42] Hands B, Parker H, Larkin D et al (2016) Male and female differences in health benefits derived from physical activity: implications for exercise prescription. J Womens Health Issues Care 5:4. 10.4172/2325-9795.1000238

[43] Allen L, Williams J, Townsend N et al (2017) Socioeconomic status and non-communicable disease behavioural risk factors in low-income and lower-middle-income countries: a systematic review. Lancet Glob Health 5:e277–e289 28193397 10.1016/S2214-109X(17)30058-X PMC5673683

[44] Monda KL, Gordon-Larsen P, Stevens J et al (2007) China’s transition: the effect of rapid urbanization on adult occupational physical activity. Soc Sci Med 64:858–870 17125897 10.1016/j.socscimed.2006.10.019 PMC2753984

[45] Sfm C, Van Cauwenberg J, Maenhout L et al (2020) Inequality in physical activity, global trends by income inequality and gender in adults. Int J Behav Nutr Phys Act 17:142 33239036 10.1186/s12966-020-01039-x PMC7690175

[46] Mayén A-L, Marques-Vidal P, Paccaud F et al (2014) Socioeconomic determinants of dietary patterns in low- and middle-income countries: a systematic review. Am J Clin Nutr 100:1520–1531 25411287 10.3945/ajcn.114.089029

[47] Haakenstad A, Yearwood JA, Fullman N et al (2022) Assessing performance of the healthcare access and quality index, overall and by select age groups, for 204 countries and territories, 1990–2019: a systematic analysis from the Global Burden of Disease Study 2019. Lancet Glob Health 10:e1715–e1743 36209761 10.1016/S2214-109X(22)00429-6 PMC9666426

[48] Neuman M, Kawachi I, Gortmaker S et al (2013) Urban-rural differences in BMI in low- and middle-income countries: the role of socioeconomic status. Am J Clin Nutr 97:428–436 23283503 10.3945/ajcn.112.045997 PMC3742298

[49] Zhang S, Sun K, Zheng R et al (2021) Cancer incidence and mortality in China, 2015. J National Cancer Center 1:2–11 10.1016/j.jncc.2020.12.001 PMC11256613 39036787

[50] Yin X, Zhang T, Zhang Y et al (2022) The global, regional, and national disease burden of breast cancer attributable to low physical activity from 1990 to 2019: an analysis of the Global Burden of Disease Study 2019. Int J Behav Nutr Phys Act 19:42 35366913 10.1186/s12966-022-01283-3 PMC8977046

[51] Ding D, Ramirez Varela A, Bauman AE et al (2020) Towards better evidence-informed global action: lessons learnt from the Lancet series and recent developments in physical activity and public health. Br J Sports Med 54:462–468 31562122 10.1136/bjsports-2019-101001 PMC7146932

[52] Molassiotis A, Kwok SWH, Leung AYM et al (2022) Associations between sociodemographic factors, health spending, disease burden, and life expectancy of older adults (70 + years old) in 22 countries in the Western Pacific Region, 1995–2019: estimates from the Global Burden of Disease (GBD) Study 2019. Geroscience 44:925–951 35000094 10.1007/s11357-021-00494-z PMC9135952

[53] Mejia-Arbelaez C, Sarmiento OL, Mora Vega R et al (2021) Social inclusion and physical activity in Ciclovía Recreativa Programs in Latin America. Int J Environ Res Public Health 18:655 33466637 10.3390/ijerph18020655 PMC7828741

[54] Pradal-Cano L, Lozano-Ruiz C, Pereyra-Rodríguez JJ et al (2020) Using mobile applications to increase physical activity: a systematic review. Int J Environ Res Public Health 17:8238 33171871 10.3390/ijerph17218238 PMC7664696

[55] World Health Organization (2020) WHO guidelines on physical activity and sedentary behaviour. https://www.who.int/publications/i/item/9789240015128. Accessed 14 Jan 2025

