# Supplementary material for: The global, regional, and national disease burden of colorectal cancer attributable to low physical activity from 1990 to 2021: an analysis of the Global Burden of Disease Study 2021
Source: Int J Colorectal Dis. 2025 Jan 18;40(1):17. doi: 10.1007/s00384-025-04811-2 (PMC11742884; doi:10.1007/s00384-025-04811-2)

**Supplementary materials**

**Supplementary Method**

1. **Estimation of colorectal cancer burden**

Colorectal cancer (CRC) was identified according to the ICD10 codes C18-C19.0, C20, C21-C21.8, Z12.1-Z12.13, Z85.03-Z85.048, and Z86.010, as well as the ICD-9 codes 153-154.9, 209.1-209.17, V10.05-V10.06, V76.41, and V76.5-V76.52 in the Global Burden of Disease Study (GBD) study.^1^ To maximize data availability, matched incidence and mortality data from cancer registries were utilized to calculate mortality-to-incidence ratios. In this way, the incidence data was converted into mortality estimation inputs.^1^ A Cause of Death Ensemble model that incorporated various sources, such as vital registration systems, cancer registries, and verbal autopsy reports was then employed to calculate the mortality data.^1^ The model provided age-, region-, and sex-specific mortality estimates by year for different cancer types.^1^ age-, region-, and sex-specific mortality estimates by year for different cancer types.^1^

Disability-adjusted life years (DALYs), for CRC were calculated by summing the years of life lost (YLL) and the years lived with disability (YLD) ^1^. The YLLs were obtained by multiplying the estimated number of CRC deaths by the GBD life expectancy for each age category, whereas the YLDs were determined by multiplying the prevalence of each condition by the corresponding disability weights for the related health state and incorporating complications arising from CRC treatment.^1^ Comprehensive input sources and metadata are available through online data tools (<https://ghdx.healthdata.org/gbd-2021/sources>).

1. **Estimation of attributable burden**

The GBD study collected self-reported data on activity frequency, duration, and intensity, lasting at least ten minutes, across various domains (leisure, work/household, and transport) from adults aged 25 and older. This was achieved through tools such as the Global Physical Activity Questionnaire, the International Physical Activity Questionnaire, and other related surveys using random sampling methods.^2^ Physical activity levels were quantified using Metabolic Equivalent (MET), representing the ratio of working metabolic rate to resting metabolic rate. Based on a prior dose-response meta-analysis, the lowest threshold to reduce physical inactivity risk was set at 3000-4500 MET-minutes per week.^3^ Therefore, activity levels below 3000 MET-minutes per week were classified as low physical activity (LPA).

A previously established Comparative Risk Assessment framework was utilized to assess the attributable burden, involving several steps.^2^ First, risk-outcome pairs with strong or likely evidence were included. Then, according to the existing systematic reviews and meta-regression, the relative risks of potential exposure were summarized. Exposure levels and distributions were estimated, and the minimum risk exposure level was defined based on published pilots and cohort studies. Population attributable fractions (PAFs) were calculated to determine the share of the CRC burden potentially reduced if the physical activity level reached 3000 MET-minutes per week.^2^ The model used for estimating PAFs was:

$$\mathrm{PA}F_{\mathrm{asgt}}=\frac{\sum_{x=1}^{u} RR_{\mathrm{ast}}(x)P_{\mathrm{asgt}}(x)-1}{\sum_{x=1}^{u} RR_{\mathrm{as}}(x)P_{\mathrm{asgt}}(x)}$$

The PAF_asgt_ represented the PAF for the CRC burden resulting from LPA, for a specific age group (a), sex (s), location (g), and year (t). The RRast denoted the relative risks for different LPA exposure levels (1 to u), while P_asgt_ was the proportion of the population exposed to LPA at level x for the corresponding age group, sex, location, and year. These relative risks were obtained from sources including both published and unpublished primary studies, as well as secondary meta-analyses, with adjustment for possible confounders made by the original researchers. Finally, after accounting for mediating effects, multiplying the total CRC burden by the PAF, allowed for the calculation of the CRC burden specifically attributable to LPA for each year, sex, age group and geographical region.^2^

**References**

1. Vos T, Lim SS, Abbafati C, et al. Global burden of 369 diseases and injuries in 204 countries and territories, 1990–2019: a systematic analysis for the Global Burden of Disease Study 2019. The Lancet 2020; 396: 1204–1222.

2. Murray CJL, Aravkin AY, Zheng P, et al. Global burden of 87 risk factors in 204 countries and territories, 1990–2019: a systematic analysis for the Global Burden of Disease Study 2019. The Lancet 2020; 396: 1223–1249.

3. Kyu HH, Bachman VF, Alexander LT, et al. Physical activity and risk of breast cancer, colon cancer, diabetes, ischemic heart disease, and ischemic stroke events: systematic review and dose-response meta-analysis for the Global Burden of Disease Study 2013. BMJ 2016; i3857.

Supplementary Table 1. Deaths of colon and rectal cancer attributable to low physical activity in 1990 and 2021 for both sexes at SDI and regional levels, with EAPC between 1990 and 2021

|  | **Male** |  |  |  |  | **Female** |  |  |  |  |
| --- | --- | --- | --- | --- | --- | --- | --- | --- | --- | --- |
|  | **Death cases in 1990**  **No. (95% *UI*)** | **ASMR per 10^5^ in 1990**  **(95% *UI*)** | **Death cases in 2021**  **No. (95% *UI*)** | **ASMR per 10^5^ in 2021**  **(95% *UI*)** | **EAPC in ASMR** | **Death cases in 1990**  **No. (95% *UI*)** | **ASMR per 10^5^ in 1990**  **(95% *UI*)** | **Death cases in 2021**  **No. (95% *UI*)** | **ASMR per 10^5^ in 2021**  **(95% *UI*)** | **EAPC in ASMR** |
| **SDI levels** |  |  |  |  |  |  |  |  |  |  |
| High SDI | 6442  (3889 to 9126) | 1.52  (0.9 to 2.15) | 10666  (6655 to 14851) | 1.07  (0.67 to 1.49) | -1.21%  (-1.26 to -1.16) | 12301  (7762 to 17145) | 1.75  (1.11 to 2.43) | 16264  (9880 to 22616) | 1.14  (0.68 to 1.57) | -1.5%  (-1.56 to -1.44) |
| High-middle SDI | 3446  (2101 to 4890) | 1.07  (0.67 to 1.54) | 8854  (5222 to 12677) | 1.12  (0.66 to 1.6) | 0.15%  (0.09 to 0.21) | 6288  (3918 to 8621) | 1.18  (0.74 to 1.62) | 11820  (7048 to 16437) | 1.02  (0.61 to 1.42) | -0.52%  (-0.59 to -0.45) |
| Low SDI | 189  (109 to 279) | 0.21  (0.12 to 0.31) | 400  (234 to 565) | 0.21  (0.12 to 0.3) | 0.13%  (-0.01 to 0.28) | 309  (170 to 464) | 0.33  (0.19 to 0.5) | 702  (433 to 998) | 0.33  (0.2 to 0.47) | -0.05%  (-0.14 to 0.04) |
| Low-middle SDI | 629  (372 to 896) | 0.25  (0.15 to 0.35) | 1907  (1169 to 2663) | 0.33  (0.2 to 0.46) | 0.9%  (0.85 to 0.96) | 1088  (655 to 1527) | 0.43  (0.26 to 0.6) | 3234  (1995 to 4508) | 0.48  (0.3 to 0.67) | 0.43%  (0.37 to 0.49) |
| Middle SDI | 2161  (1302 to 3167) | 0.58  (0.35 to 0.85) | 7975  (4787 to 11623) | 0.74  (0.45 to 1.08) | 0.75%  (0.7 to 0.79) | 3593  (2160 to 5099) | 0.8  (0.49 to 1.13) | 9558  (5927 to 13572) | 0.72  (0.44 to 1.02) | -0.51%  (-0.57 to -0.45) |
| **Region levels** |  |  |  |  |  |  |  |  |  |  |
| Andean Latin America | 25  (14 to 40) | 0.29  (0.16 to 0.47) | 92  (48 to 146) | 0.35  (0.18 to 0.57) | 0.89%  (0.79 to 0.99) | 49  (26 to 74) | 0.52  (0.28 to 0.8) | 175  (96 to 273) | 0.58  (0.32 to 0.91) | 0.33%  (0.19 to 0.47) |
| Australasia | 198  (114 to 298) | 2.08  (1.16 to 3.13) | 354  (194 to 534) | 1.34  (0.74 to 2.03) | -1.62%  (-1.74 to -1.49) | 320  (189 to 448) | 2.35  (1.4 to 3.27) | 478  (289 to 719) | 1.45  (0.88 to 2.15) | -1.64%  (-1.7 to -1.58) |
| Caribbean | 64  (34 to 97) | 0.58  (0.31 to 0.87) | 157  (91 to 240) | 0.65  (0.37 to 0.99) | 0.47%  (0.41 to 0.52) | 172  (104 to 238) | 1.36  (0.82 to 1.88) | 403  (239 to 594) | 1.35  (0.81 to 1.97) | 0.05%  (0 to 0.09) |
| Central Asia | 52  (30 to 74) | 0.34  (0.2 to 0.48) | 83  (48 to 121) | 0.32  (0.18 to 0.46) | 0.47%  (0.21 to 0.73) | 126  (75 to 179) | 0.46  (0.27 to 0.65) | 153  (96 to 217) | 0.37  (0.23 to 0.53) | -0.17%  (-0.35 to 0.02) |
| Central Europe | 610  (377 to 845) | 1.12  (0.69 to 1.56) | 1405  (842 to 1988) | 1.52  (0.91 to 2.16) | 1%  (0.91 to 1.1) | 1265  (789 to 1755) | 1.48  (0.92 to 2.06) | 2098  (1296 to 2935) | 1.42  (0.88 to 1.99) | -0.36%  (-0.5 to -0.23) |
| Central Latin America | 78  (43 to 116) | 0.23  (0.12 to 0.35) | 387  (227 to 563) | 0.36  (0.21 to 0.52) | 1.46%  (1.17 to 1.74) | 194  (113 to 273) | 0.52  (0.3 to 0.74) | 755  (474 to 1060) | 0.57  (0.36 to 0.8) | 0.35%  (0.15 to 0.54) |
| Central Sub-Saharan Africa | 28  (16 to 45) | 0.39  (0.21 to 0.62) | 62  (33 to 103) | 0.41  (0.21 to 0.66) | 0.16%  (-0.07 to 0.39) | 29  (16 to 46) | 0.33  (0.19 to 0.53) | 81  (43 to 135) | 0.35  (0.19 to 0.62) | 0.18%  (0.08 to 0.28) |
| East Asia | 2328  (1328 to 3706) | 0.87  (0.46 to 1.39) | 8606  (4664 to 13685) | 1.01  (0.53 to 1.61) | 0.52%  (0.45 to 0.59) | 3653  (2062 to 5420) | 1  (0.56 to 1.51) | 8956  (4954 to 13539) | 0.81  (0.44 to 1.22) | -0.91%  (-1.01 to -0.8) |
| Eastern Europe | 624  (358 to 902) | 0.84  (0.47 to 1.22) | 1194  (705 to 1834) | 1.03  (0.59 to 1.58) | 0.59%  (0.45 to 0.73) | 1694  (968 to 2365) | 0.9  (0.52 to 1.25) | 2517  (1467 to 3634) | 1.01  (0.59 to 1.45) | 0.27%  (0.2 to 0.34) |
| Eastern Sub-Saharan Africa | 64  (32 to 106) | 0.21  (0.1 to 0.34) | 143  (78 to 215) | 0.25  (0.13 to 0.42) | 0.58%  (0.45 to 0.7) | 93  (50 to 144) | 0.3  (0.16 to 0.45) | 218  (126 to 322) | 0.3  (0.17 to 0.44) | -0.03%  (-0.14 to 0.08) |
| High-income Asia Pacific | 1151  (587 to 1721) | 1.48  (0.74 to 2.3) | 2782  (1463 to 4459) | 1.24  (0.66 to 1.92) | -0.69%  (-0.75 to -0.63) | 1531  (924 to 2183) | 1.37  (0.82 to 1.97) | 3921  (1952 to 6127) | 1.09  (0.62 to 1.64) | -0.79%  (-0.85 to -0.74) |
| High-income North America | 1417  (696 to 2467) | 1.02  (0.5 to 1.76) | 1908  (1021 to 3017) | 0.64  (0.34 to 1) | -1.7%  (-1.87 to -1.54) | 3176  (1793 to 4666) | 1.36  (0.77 to 1.98) | 3710  (2139 to 5412) | 0.91  (0.53 to 1.31) | -1.43%  (-1.51 to -1.35) |
| North Africa and Middle East | 399  (237 to 562) | 0.58  (0.35 to 0.84) | 1235  (720 to 1778) | 0.65  (0.38 to 0.95) | 0.5%  (0.38 to 0.63) | 674  (402 to 970) | 0.95  (0.57 to 1.35) | 1803  (1104 to 2528) | 0.93  (0.58 to 1.3) | 0.3%  (0.11 to 0.49) |
| Oceania | 3  (2 to 4) | 0.24  (0.14 to 0.35) | 6  (4 to 9) | 0.2  (0.12 to 0.3) | -0.56%  (-0.68 to -0.44) | 7  (4 to 11) | 0.57  (0.34 to 0.84) | 16  (10 to 23) | 0.48  (0.29 to 0.71) | -0.51%  (-0.59 to -0.44) |
| South Asia | 523  (293 to 775) | 0.21  (0.11 to 0.3) | 1474  (879 to 2227) | 0.24  (0.14 to 0.37) | 0.21%  (0.07 to 0.34) | 702  (401 to 1045) | 0.31  (0.18 to 0.46) | 2114  (1335 to 3039) | 0.32  (0.2 to 0.45) | -0.09%  (-0.22 to 0.05) |
| Southeast Asia | 566  (328 to 819) | 0.61  (0.35 to 0.87) | 2314  (1364 to 3399) | 0.93  (0.55 to 1.37) | 1.33%  (1.27 to 1.39) | 934  (547 to 1359) | 0.79  (0.46 to 1.14) | 2951  (1803 to 4325) | 0.93  (0.57 to 1.36) | 0.43%  (0.32 to 0.54) |
| Southern Latin America | 103  (54 to 172) | 0.58  (0.3 to 1.04) | 209  (101 to 353) | 0.57  (0.28 to 0.97) | 0.29%  (0.16 to 0.43) | 233  (134 to 365) | 0.93  (0.53 to 1.46) | 454  (257 to 675) | 0.83  (0.47 to 1.22) | -0.14%  (-0.29 to 0.01) |
| Southern Sub-Saharan Africa | 76  (43 to 116) | 0.81  (0.46 to 1.22) | 206  (118 to 293) | 1.1  (0.63 to 1.57) | 0.92%  (0.65 to 1.19) | 116  (66 to 183) | 0.85  (0.48 to 1.34) | 314  (191 to 443) | 1.07  (0.65 to 1.53) | 0.76%  (0.58 to 0.95) |
| Tropical Latin America | 230  (134 to 338) | 0.7  (0.4 to 1.02) | 989  (557 to 1463) | 0.94  (0.53 to 1.39) | 1.21%  (1.12 to 1.31) | 417  (245 to 595) | 1  (0.6 to 1.44) | 1577  (924 to 2271) | 1.11  (0.65 to 1.59) | 0.35%  (0.24 to 0.45) |
| Western Europe | 4275  (2575 to 6005) | 1.91  (1.15 to 2.7) | 6072  (3702 to 8549) | 1.31  (0.8 to 1.83) | -1.26%  (-1.31 to -1.22) | 8123  (5115 to 11164) | 2.09  (1.32 to 2.88) | 8660  (5286 to 12051) | 1.28  (0.79 to 1.77) | -1.67%  (-1.73 to -1.62) |
| Western Sub-Saharan Africa | 68  (38 to 102) | 0.19  (0.11 to 0.29) | 160  (88 to 234) | 0.22  (0.12 to 0.33) | 0.69%  (0.58 to 0.8) | 107  (64 to 156) | 0.29  (0.17 to 0.42) | 282  (163 to 404) | 0.35  (0.2 to 0.51) | 0.82%  (0.75 to 0.9) |

Supplementary Table 2. DALYs of colon and rectal cancer attributable to low physical activity in 1990 and 2021 for both sexes at SDI and regional levels, with EAPC between 1990 and 2021

|  | **Male** |  |  |  |  | **Female** |  |  |  |  |
| --- | --- | --- | --- | --- | --- | --- | --- | --- | --- | --- |
|  | **DALYs in 1990**  **No. (95% UI)** | **ASDR per 10^5^ in 1990**  **(95% UI)** | **DALYs in 2021**  **No. (95% UI)** | **ASDR per 10^5^ in 2021**  **(95% UI)** | **EAPC in ASDR** | **DALYs in 1990**  **No. (95% UI)** | **ASDR per 10^5^ in 1990**  **(95% UI)** | **DALYs in 2021**  **No. (95% UI)** | **ASDR per 10^5^ in 2021**  **(95% UI)** | **EAPC in ASDR** |
| **SDI levels** |  |  |  |  |  |  |  |  |  |  |
| High SDI | 127614  (77165 to 177629) | 28.1  (16.97 to 39.28) | 191088  (119081 to 263450) | 19.81  (12.38 to 27.22) | -1.24%  (-1.3 to -1.18) | 215859  (136231 to 296678) | 32.55  (20.48 to 44.54) | 257533  (155547 to 355615) | 21.16  (13.08 to 29.31) | -1.48%  (-1.56 to -1.41) |
| High-middle SDI | 70846  (42523 to 102178) | 18.8  (11.36 to 26.59) | 165582  (99809 to 239172) | 19.45  (11.76 to 27.87) | 0.1%  (0.07 to 0.13) | 124436  (77935 to 170828) | 22.37  (13.94 to 30.66) | 209674  (125128 to 291410) | 18.74  (11.23 to 26.08) | -0.69%  (-0.74 to -0.64) |
| Low SDI | 4467  (2578 to 6660) | 4.17  (2.42 to 6.18) | 8906  (5363 to 12464) | 4.01  (2.35 to 5.63) | -0.18%  (-0.33 to -0.04) | 7739  (4054 to 11795) | 7.14  (3.86 to 10.81) | 16761  (10139 to 24023) | 6.68  (4.12 to 9.48) | -0.28%  (-0.36 to -0.2) |
| Low-middle SDI | 14952  (8874 to 21387) | 5.09  (3.01 to 7.26) | 42246  (26373 to 58936) | 6.46  (4 to 8.99) | 0.68%  (0.63 to 0.73) | 26340  (15909 to 37494) | 9  (5.45 to 12.69) | 72580  (44196 to 102839) | 9.89  (6.06 to 13.93) | 0.3%  (0.26 to 0.34) |
| Middle SDI | 49897  (29778 to 71491) | 11.03  (6.63 to 16.12) | 165876  (98469 to 243562) | 13.78  (8.35 to 20.16) | 0.68%  (0.61 to 0.74) | 82113  (48769 to 116939) | 16.05  (9.58 to 22.74) | 197720  (123723 to 276514) | 14.17  (8.86 to 19.72) | -0.56%  (-0.64 to -0.49) |
| **Regions** |  |  |  |  |  |  |  |  |  |  |
| Andean Latin America | 522  (296 to 833) | 5.56  (3.17 to 8.92) | 1840  (986 to 2875) | 6.72  (3.51 to 10.61) | 0.83%  (0.71 to 0.94) | 907  (475 to 1402) | 9.16  (4.84 to 14.14) | 3139  (1800 to 4772) | 10.3  (5.89 to 15.68) | 0.32%  (0.2 to 0.45) |
| Australasia | 4140  (2390 to 6166) | 40.24  (23.03 to 60.55) | 6473  (3621 to 9617) | 25.64  (14.55 to 38.55) | -1.68%  (-1.81 to -1.55) | 6176  (3691 to 8681) | 47.42  (28.55 to 65.98) | 8231  (5030 to 12015) | 28.54  (17.54 to 40.77) | -1.71%  (-1.77 to -1.64) |
| Caribbean | 1230  (659 to 1801) | 10.29  (5.51 to 15.1) | 3010  (1724 to 4443) | 12.13  (6.99 to 17.89) | 0.67%  (0.61 to 0.72) | 3453  (2081 to 4821) | 26.11  (15.74 to 36.68) | 7718  (4677 to 11226) | 26.65  (16.17 to 38.75) | 0.15%  (0.11 to 0.19) |
| Central Asia | 1158  (647 to 1703) | 6.76  (3.89 to 9.65) | 1744  (1010 to 2584) | 5.79  (3.36 to 8.44) | 0.12%  (-0.11 to 0.35) | 2764  (1663 to 3926) | 9.91  (5.93 to 14.1) | 3262  (1989 to 4556) | 7.37  (4.51 to 10.34) | -0.6%  (-0.72 to -0.47) |
| Central Europe | 12083  (7304 to 16741) | 20.17  (12.3 to 27.9) | 25646  (15342 to 36219) | 26.81  (16.22 to 37.95) | 0.95%  (0.87 to 1.03) | 23996  (14993 to 33239) | 27.73  (17.17 to 38.53) | 35438  (21724 to 48944) | 26.19  (16.04 to 36.17) | -0.37%  (-0.49 to -0.25) |
| Central Latin America | 1695  (966 to 2500) | 4.45  (2.46 to 6.53) | 8719  (5163 to 12771) | 7.62  (4.46 to 11.12) | 1.82%  (1.49 to 2.15) | 4156  (2454 to 5769) | 9.99  (5.8 to 13.88) | 16593  (10239 to 23657) | 12.22  (7.57 to 17.31) | 0.73%  (0.5 to 0.95) |
| Central Sub-Saharan Africa | 630  (352 to 1011) | 7.18  (4.03 to 11.38) | 1422  (728 to 2396) | 7.35  (3.9 to 12.03) | 0.07%  (-0.18 to 0.32) | 739  (416 to 1153) | 6.72  (3.83 to 10.43) | 1927  (1021 to 3127) | 6.96  (3.74 to 11.67) | 0.12%  (0.01 to 0.23) |
| East Asia | 52854  (29352 to 84892) | 15.1  (8.5 to 23.71) | 167329  (90318 to 271419) | 17.36  (9.33 to 27.41) | 0.48%  (0.38 to 0.58) | 81732  (45222 to 124131) | 19.37  (10.99 to 28.9) | 170829  (94040 to 258978) | 15.03  (8.41 to 22.55) | -1.04%  (-1.15 to -0.93) |
| Eastern Europe | 13087  (7447 to 19057) | 15.19  (8.64 to 21.86) | 22370  (13105 to 33953) | 17.84  (10.47 to 26.94) | 0.43%  (0.31 to 0.55) | 33914  (19756 to 47784) | 18.22  (10.78 to 25.47) | 43787  (25290 to 62307) | 18.68  (10.58 to 26.52) | -0.13%  (-0.21 to -0.05) |
| Eastern Sub-Saharan Africa | 1537  (767 to 2549) | 4.33  (2.19 to 7.14) | 3187  (1791 to 4885) | 4.6  (2.52 to 7) | 0.11%  (-0.04 to 0.26) | 2405  (1268 to 3734) | 6.47  (3.45 to 10.03) | 5258  (3009 to 7654) | 6.11  (3.53 to 8.99) | -0.3%  (-0.41 to -0.19) |
| High-income Asia Pacific | 27623  (14931 to 39870) | 31.6  (16.64 to 45.79) | 51754  (28361 to 77892) | 25.51  (14.19 to 37.68) | -0.8%  (-0.86 to -0.73) | 33118  (20365 to 47321) | 29.39  (17.94 to 41.77) | 58465  (32996 to 88071) | 21.6  (12.06 to 31.31) | -1.07%  (-1.12 to -1.02) |
| High-income North America | 25760  (12848 to 43330) | 17.64  (8.73 to 30.05) | 34260  (18690 to 52062) | 11.4  (6.23 to 17.53) | -1.61%  (-1.86 to -1.35) | 53772  (30898 to 77810) | 24.78  (14.16 to 35.95) | 63407  (37493 to 91954) | 17.31  (10.17 to 25.04) | -1.22%  (-1.36 to -1.08) |
| North Africa and Middle East | 9595  (5834 to 13545) | 11.78  (7.03 to 16.6) | 28316  (16672 to 40222) | 12.88  (7.58 to 18.39) | 0.42%  (0.3 to 0.54) | 16922  (9895 to 24618) | 20.33  (12.01 to 29.35) | 43348  (25890 to 61805) | 19.35  (11.73 to 27.09) | 0.09%  (-0.06 to 0.23) |
| Oceania | 79  (46 to 118) | 5.19  (3.11 to 7.57) | 172  (97 to 256) | 4.38  (2.55 to 6.5) | -0.59%  (-0.68 to -0.5) | 211  (121 to 327) | 13.69  (7.92 to 20.66) | 470  (278 to 697) | 11.7  (7 to 17.17) | -0.52%  (-0.58 to -0.46) |
| South Asia | 13072  (7269 to 19378) | 4.46  (2.5 to 6.57) | 32774  (19694 to 49783) | 4.73  (2.84 to 7.13) | -0.19%  (-0.35 to -0.04) | 16993  (9745 to 25830) | 6.49  (3.72 to 9.72) | 45491  (28217 to 65843) | 6.24  (3.91 to 9.02) | -0.32%  (-0.45 to -0.19) |
| Southeast Asia | 12998  (7533 to 19145) | 11.91  (6.9 to 17.36) | 51891  (29806 to 77589) | 18.19  (10.6 to 26.91) | 1.33%  (1.28 to 1.37) | 22206  (12757 to 32739) | 16.77  (9.7 to 24.6) | 64758  (38845 to 97726) | 18.74  (11.41 to 27.91) | 0.26%  (0.17 to 0.34) |
| Southern Latin America | 2051  (1110 to 3418) | 10.57  (5.69 to 17.44) | 3803  (1951 to 6180) | 10.09  (5.16 to 16.54) | 0.12%  (0.03 to 0.21) | 4251  (2522 to 6410) | 16.61  (9.88 to 24.92) | 7673  (4322 to 11394) | 15.08  (8.53 to 22.37) | -0.11%  (-0.2 to -0.02) |
| Southern Sub-Saharan Africa | 1819  (1045 to 2767) | 16.06  (9.05 to 24.63) | 4983  (2936 to 7139) | 21.68  (12.6 to 30.98) | 0.88%  (0.59 to 1.16) | 2471  (1428 to 3836) | 16.42  (9.47 to 25.55) | 6743  (4004 to 9602) | 20.83  (12.56 to 29.49) | 0.93%  (0.77 to 1.09) |
| Tropical Latin America | 4840  (2779 to 7186) | 12.64  (7.31 to 18.39) | 20478  (11436 to 30257) | 18.22  (10.25 to 26.8) | 1.39%  (1.3 to 1.47) | 8738  (5139 to 12522) | 18.92  (11.07 to 26.62) | 32124  (19005 to 46560) | 22.64  (13.38 to 32.77) | 0.58%  (0.49 to 0.66) |
| Western Europe | 79733  (48303 to 111541) | 33.83  (20.61 to 47.26) | 100593  (61192 to 140466) | 22.93  (14.06 to 31.96) | -1.32%  (-1.37 to -1.28) | 135866  (85053 to 187911) | 37.74  (23.51 to 52.35) | 130267  (82147 to 180436) | 22.98  (14.33 to 31.63) | -1.68%  (-1.73 to -1.62) |
| Western Sub-Saharan Africa | 1602  (912 to 2418) | 3.82  (2.16 to 5.73) | 3607  (2016 to 5173) | 4.25  (2.37 to 6.21) | 0.51%  (0.41 to 0.61) | 2365  (1403 to 3465) | 5.74  (3.43 to 8.37) | 6390  (3646 to 9388) | 6.62  (3.81 to 9.54) | 0.64%  (0.56 to 0.72) |

Supplementary Table 3. Deaths and DALYs of colon and rectal cancer attributable to low physical activity in 1990 and 2021 at regional levels, with EAPC between 1990 and 2021.

|  | **1990** |  |  |  | **2021** |  |  |  | **EAPC** |  |
| --- | --- | --- | --- | --- | --- | --- | --- | --- | --- | --- |
| **Regions** | **Death cases in 1990**  **No. (95% *UI*)** | **ASMR per 10^5^ in 1990**  **(95% *UI*)** | **DALYs in 1990**  **No. (95% UI)** | **ASDR per 10^5^ in 1990**  **(95% UI)** | **Death cases in 2021**  **No. (95% *UI*)** | **ASMR per 10^5^ in 2021**  **No. (95% *UI*)** | **DALYs in 2021**  **No. (95% UI)** | **ASDR per 10^5^ in 2021**  **(95% UI)** | **EAPC in ASMR**  **(1990-2021)** | **EAPC in ASDR**  **(1990-2021)** |
| Andean Latin America | 73  (42 to 108) | 0.41  (0.24 to 0.61) | 1430  (797 to 2063) | 7.46  (4.19 to 10.82) | 267  (149 to 406) | 0.48  (0.27 to 0.73) | 4979  (2833 to 7577) | 8.64  (4.87 to 13.19) | 0.51%  (0.4 to 0.62) | 0.5%  (0.4 to 0.6) |
| Australasia | 518  (309 to 714) | 2.23  (1.33 to 3.09) | 10316  (6277 to 14337) | 44.01  (26.85 to 61.19) | 832  (503 to 1172) | 1.41  (0.86 to 1.97) | 14704  (9018 to 20648) | 27.18  (16.7 to 38.35) | -1.63%  (-1.72 to -1.55) | -1.69%  (-1.78 to -1.61) |
| Caribbean | 236  (140 to 325) | 0.99  (0.58 to 1.39) | 4683  (2785 to 6433) | 18.55  (11.05 to 25.45) | 560  (336 to 822) | 1.03  (0.62 to 1.51) | 10727  (6420 to 15253) | 19.88  (11.94 to 28.26) | 0.2%  (0.15 to 0.24) | 0.32%  (0.28 to 0.35) |
| Central Asia | 178  (106 to 249) | 0.41  (0.24 to 0.58) | 3922  (2390 to 5522) | 8.58  (5.18 to 12.06) | 237  (146 to 332) | 0.35  (0.22 to 0.49) | 5006  (3019 to 7015) | 6.64  (4.02 to 9.3) | 0.05%  (-0.17 to 0.26) | -0.36%  (-0.52 to -0.19) |
| Central Europe | 1875  (1165 to 2586) | 1.34  (0.82 to 1.84) | 36079  (22597 to 49321) | 24.51  (15.28 to 33.6) | 3503  (2164 to 4843) | 1.45  (0.9 to 2.01) | 61083  (37459 to 84090) | 26.26  (15.99 to 36.25) | 0.11%  (-0.01 to 0.22) | 0.1%  (0 to 0.2) |
| Central Latin America | 273  (164 to 374) | 0.38  (0.23 to 0.53) | 5851  (3539 to 8083) | 7.33  (4.41 to 10.15) | 1142  (699 to 1616) | 0.47  (0.29 to 0.67) | 25312  (15516 to 35384) | 10.11  (6.22 to 14.15) | 0.74%  (0.52 to 0.96) | 1.11%  (0.85 to 1.36) |
| Central Sub-Saharan Africa | 57  (34 to 86) | 0.36  (0.21 to 0.56) | 1369  (798 to 2034) | 6.95  (4.14 to 10.38) | 143  (81 to 224) | 0.37  (0.21 to 0.6) | 3349  (1902 to 5131) | 7.03  (3.98 to 11.08) | 0.1%  (-0.06 to 0.27) | 0.04%  (-0.13 to 0.22) |
| East Asia | 5981  (3504 to 8704) | 0.93  (0.55 to 1.35) | 134586  (78500 to 196219) | 17.19  (10.11 to 24.87) | 17562  (10720 to 25680) | 0.88  (0.54 to 1.29) | 338157  (205644 to 497747) | 15.92  (9.73 to 23.12) | -0.27%  (-0.35 to -0.2) | -0.36%  (-0.46 to -0.26) |
| Eastern Europe | 2318  (1364 to 3145) | 0.87  (0.52 to 1.18) | 47001  (27683 to 64804) | 16.85  (9.83 to 23.02) | 3712  (2187 to 5326) | 1.01  (0.6 to 1.45) | 66157  (38265 to 95460) | 18.15  (10.5 to 26.11) | 0.43%  (0.35 to 0.51) | 0.1%  (0.02 to 0.18) |
| Eastern Sub-Saharan Africa | 157  (89 to 235) | 0.25  (0.14 to 0.38) | 3942  (2196 to 5828) | 5.42  (3.05 to 8.07) | 361  (217 to 529) | 0.28  (0.16 to 0.42) | 8445  (5065 to 11882) | 5.38  (3.23 to 7.82) | 0.22%  (0.11 to 0.34) | -0.13%  (-0.25 to 0) |
| High-income Asia Pacific | 2682  (1601 to 3800) | 1.41  (0.84 to 2.01) | 60740  (36391 to 84196) | 30.27  (17.98 to 42.07) | 6703  (3824 to 9944) | 1.17  (0.68 to 1.68) | 110219  (64737 to 156895) | 23.51  (14.19 to 32.82) | -0.69%  (-0.73 to -0.64) | -0.9%  (-0.95 to -0.85) |
| High-income North America | 4594  (2672 to 6907) | 1.23  (0.71 to 1.84) | 79531  (46858 to 116595) | 21.74  (12.65 to 31.46) | 5618  (3320 to 8008) | 0.79  (0.47 to 1.13) | 97667  (57378 to 139663) | 14.6  (8.5 to 20.8) | -1.58%  (-1.68 to -1.47) | -1.41%  (-1.58 to -1.23) |
| North Africa and Middle East | 1073  (676 to 1489) | 0.77  (0.48 to 1.06) | 26518  (16267 to 37376) | 16.02  (10.02 to 22.3) | 3039  (1892 to 4197) | 0.79  (0.5 to 1.1) | 71664  (43790 to 99243) | 16.05  (9.92 to 22.05) | 0.37%  (0.2 to 0.54) | 0.21%  (0.07 to 0.35) |
| Oceania | 10  (6 to 15) | 0.4  (0.24 to 0.58) | 291  (172 to 436) | 9.32  (5.6 to 13.78) | 22  (13 to 33) | 0.34  (0.2 to 0.49) | 642  (382 to 942) | 7.92  (4.79 to 11.64) | -0.56%  (-0.65 to -0.47) | -0.56%  (-0.63 to -0.49) |
| South Asia | 1225  (747 to 1716) | 0.26  (0.15 to 0.36) | 30066  (18294 to 42154) | 5.44  (3.31 to 7.61) | 3588  (2223 to 5062) | 0.28  (0.17 to 0.39) | 78265  (48180 to 111435) | 5.52  (3.42 to 7.8) | 0.09%  (-0.02 to 0.21) | -0.22%  (-0.35 to -0.09) |
| Southeast Asia | 1499  (909 to 2166) | 0.71  (0.43 to 1.01) | 35203  (21190 to 50985) | 14.48  (8.77 to 20.85) | 5265  (3099 to 7363) | 0.93  (0.55 to 1.31) | 116649  (67993 to 164520) | 18.44  (10.85 to 25.77) | 0.81%  (0.72 to 0.9) | 0.7%  (0.63 to 0.77) |
| Southern Latin America | 336  (207 to 503) | 0.79  (0.48 to 1.2) | 6301  (3850 to 9205) | 14.05  (8.7 to 20.6) | 663  (380 to 993) | 0.73  (0.42 to 1.09) | 11477  (6490 to 16897) | 12.9  (7.29 to 18.92) | -0.02%  (-0.17 to 0.12) | -0.06%  (-0.15 to 0.03) |
| Southern Sub-Saharan Africa | 192  (112 to 283) | 0.84  (0.49 to 1.26) | 4290  (2514 to 6367) | 16.31  (9.59 to 24.18) | 520  (324 to 715) | 1.09  (0.67 to 1.49) | 11726  (7113 to 16042) | 21.18  (13.05 to 29.11) | 0.8%  (0.58 to 1.02) | 0.88%  (0.67 to 1.09) |
| Tropical Latin America | 647  (387 to 897) | 0.87  (0.52 to 1.2) | 13578  (8223 to 19088) | 16.03  (9.55 to 22.31) | 2566  (1525 to 3618) | 1.04  (0.62 to 1.46) | 52602  (31235 to 74188) | 20.6  (12.27 to 28.95) | 0.67%  (0.6 to 0.75) | 0.88%  (0.82 to 0.94) |
| Western Europe | 12399  (7760 to 17073) | 2.02  (1.27 to 2.8) | 215599  (134307 to 296195) | 36.03  (22.47 to 49.55) | 14731  (9242 to 20357) | 1.29  (0.81 to 1.78) | 230860  (144787 to 318510) | 22.91  (14.23 to 31.63) | -1.52%  (-1.57 to -1.47) | -1.53%  (-1.58 to -1.49) |
| Western Sub-Saharan Africa | 175  (107 to 255) | 0.24  (0.15 to 0.35) | 3967  (2398 to 5743) | 4.8  (2.91 to 6.99) | 441  (257 to 617) | 0.29  (0.17 to 0.41) | 9997  (5764 to 14150) | 5.51  (3.22 to 7.72) | 0.76%  (0.68 to 0.85) | 0.61%  (0.52 to 0.7) |

**Supplementary Table 4. Deaths and DALYs of colon and rectal cancer attributable to low physical activity in 1990 and 2021 for both sexes in 204 countries and territories, with EAPC between 1990 and 2021**

|  | **1990** |  |  |  | **2021** |  |  |  | **EAPC** |  |
| --- | --- | --- | --- | --- | --- | --- | --- | --- | --- | --- |
|  | **Death cases in 1990**  **No. (95% *UI*)** | **ASMR per 10^5^ in 1990**  **(95% *UI*)** | **DALYs in 1990**  **No. (95% UI)** | **ASDR per 10^5^ in 1990**  **(95% UI)** | **Death cases in 2021**  **No. (95% *UI*)** | **ASMR per 10^5^ in 2021**  **(95% *UI*)** | **DALYs in 2021**  **No. (95% *UI*)** | **ASDR per 10^5^ in 2021**  **(95% *UI*)** | **EAPC in ASMR** | **EAPC in ASDR** |
| Afghanistan | 64  (20 to 114) | 1.01  (0.36 to 1.76) | 1715  (480 to 3112) | 24.31  (7.44 to 43.87) | 97  (38 to 170) | 1.12  (0.48 to 1.86) | 2833  (960 to 5027) | 26.5  (10.16 to 45.81) | 0.52%  (0.43 to 0.61) | 0.42%  (0.34 to 0.5) |
| Albania | 7  (4 to 11) | 0.42  (0.23 to 0.64) | 133  (74 to 202) | 7.24  (4.03 to 10.98) | 19  (11 to 30) | 0.45  (0.25 to 0.71) | 328  (181 to 516) | 7.42  (4.12 to 11.55) | 0.63%  (0.41 to 0.85) | 0.47%  (0.25 to 0.69) |
| Algeria | 42  (24 to 62) | 0.5  (0.28 to 0.75) | 982  (548 to 1406) | 9.11  (5.26 to 13.21) | 127  (75 to 193) | 0.46  (0.26 to 0.69) | 2747  (1599 to 4128) | 8.29  (4.9 to 12.51) | 0.25%  (0.05 to 0.45) | -0.03%  (-0.16 to 0.11) |
| American Samoa | 0  (0 to 0) | 1.6  (0.99 to 2.27) | 9  (6 to 13) | 37.93  (23.74 to 53.71) | 1  (0 to 1) | 1.64  (0.98 to 2.48) | 19  (12 to 29) | 39.19  (23.33 to 58.2) | 0.2%  (0.11 to 0.28) | 0.22%  (0.14 to 0.31) |
| Andorra | 1  (0 to 2) | 1.67  (0.84 to 2.89) | 17  (9 to 29) | 30.24  (15.27 to 51.89) | 2  (1 to 3) | 1.11  (0.61 to 1.83) | 31  (17 to 51) | 19.65  (10.69 to 32.15) | -1.03%  (-1.25 to -0.81) | -1.1%  (-1.32 to -0.88) |
| Angola | 9  (5 to 14) | 0.31  (0.17 to 0.5) | 223  (115 to 366) | 6.31  (3.43 to 10.1) | 30  (16 to 50) | 0.36  (0.19 to 0.61) | 738  (380 to 1225) | 6.98  (3.81 to 11.69) | 0.47%  (0.38 to 0.55) | 0.33%  (0.24 to 0.41) |
| Antigua and Barbuda | 1  (0 to 1) | 0.87  (0.49 to 1.28) | 9  (5 to 13) | 15.77  (9.2 to 23.15) | 1  (1 to 2) | 1.14  (0.64 to 1.66) | 21  (12 to 31) | 20.51  (11.96 to 30.39) | 0.94%  (0.73 to 1.14) | 0.86%  (0.67 to 1.05) |
| Argentina | 237  (136 to 366) | 0.8  (0.46 to 1.27) | 4369  (2552 to 6693) | 13.98  (8.38 to 21.19) | 427  (230 to 689) | 0.73  (0.39 to 1.17) | 7257  (4016 to 11389) | 12.69  (7.01 to 19.83) | 0.02%  (-0.16 to 0.2) | -0.04%  (-0.17 to 0.08) |
| Armenia | 14  (8 to 21) | 0.59  (0.33 to 0.87) | 294  (172 to 443) | 11.45  (6.55 to 16.74) | 30  (17 to 46) | 0.7  (0.39 to 1.03) | 531  (288 to 798) | 12.22  (6.82 to 18.07) | 1.17%  (0.92 to 1.41) | 0.68%  (0.46 to 0.89) |
| Australia | 427  (250 to 601) | 2.21  (1.3 to 3.12) | 8715  (5177 to 12194) | 44.71  (26.57 to 62.56) | 668  (396 to 964) | 1.34  (0.81 to 1.92) | 12065  (7517 to 17145) | 26.77  (16.45 to 38.47) | -1.83%  (-1.93 to -1.73) | -1.87%  (-1.97 to -1.76) |
| Austria | 247  (144 to 361) | 1.92  (1.12 to 2.79) | 4148  (2387 to 6031) | 33.29  (19.37 to 47.68) | 191  (106 to 304) | 0.87  (0.49 to 1.36) | 2932  (1681 to 4545) | 14.75  (8.54 to 22.95) | -2.68%  (-2.76 to -2.59) | -2.75%  (-2.84 to -2.66) |
| Azerbaijan | 16  (8 to 23) | 0.36  (0.2 to 0.53) | 340  (177 to 516) | 7.21  (3.84 to 10.68) | 26  (15 to 44) | 0.33  (0.18 to 0.53) | 574  (312 to 957) | 6.23  (3.43 to 10.33) | 0.39%  (0.03 to 0.75) | 0.05%  (-0.24 to 0.33) |
| Bahamas | 1  (1 to 2) | 0.97  (0.6 to 1.44) | 30  (18 to 43) | 19.7  (11.87 to 29.03) | 4  (2 to 7) | 1.22  (0.65 to 1.91) | 91  (51 to 146) | 23.43  (12.73 to 37.18) | 0.97%  (0.77 to 1.16) | 0.85%  (0.7 to 1.01) |
| Bahrain | 1  (1 to 2) | 1.22  (0.66 to 1.77) | 38  (22 to 54) | 23.57  (13.36 to 33.67) | 6  (3 to 9) | 1.09  (0.61 to 1.66) | 151  (84 to 234) | 19.59  (11.02 to 29.75) | -0.64%  (-0.98 to -0.3) | -0.99%  (-1.27 to -0.71) |
| Bangladesh | 71  (41 to 114) | 0.17  (0.1 to 0.26) | 1761  (996 to 2861) | 3.75  (2.15 to 6.08) | 182  (98 to 303) | 0.14  (0.08 to 0.24) | 4256  (2254 to 7216) | 3.09  (1.65 to 5.2) | -0.46%  (-0.59 to -0.34) | -0.56%  (-0.7 to -0.41) |
| Barbados | 6  (4 to 8) | 1.91  (1.18 to 2.65) | 111  (69 to 155) | 38.07  (23.52 to 52.95) | 13  (7 to 19) | 2.41  (1.38 to 3.56) | 241  (141 to 358) | 46.69  (27.04 to 69.32) | 1.17%  (0.82 to 1.52) | 1.14%  (0.82 to 1.46) |
| Belarus | 103  (59 to 151) | 0.81  (0.46 to 1.17) | 2055  (1169 to 3054) | 15.87  (9.01 to 23.4) | 156  (87 to 248) | 0.95  (0.53 to 1.48) | 2925  (1631 to 4611) | 17.82  (10.02 to 27.57) | -0.11%  (-0.46 to 0.23) | -0.28%  (-0.61 to 0.06) |
| Belgium | 369  (217 to 526) | 2.27  (1.34 to 3.25) | 6061  (3517 to 8768) | 37.94  (21.88 to 54.65) | 339  (192 to 514) | 1.18  (0.67 to 1.75) | 5160  (2951 to 7623) | 20.12  (11.49 to 29.48) | -2.08%  (-2.16 to -1.99) | -2.07%  (-2.15 to -1.99) |
| Belize | 0  (0 to 1) | 0.41  (0.24 to 0.58) | 7  (4 to 10) | 7.75  (4.56 to 11.03) | 2  (1 to 2) | 0.59  (0.32 to 0.86) | 33  (19 to 50) | 11.47  (6.41 to 17.33) | 1.22%  (0.75 to 1.7) | 1.29%  (0.85 to 1.72) |
| Benin | 2  (1 to 3) | 0.1  (0.05 to 0.18) | 33  (18 to 55) | 1.83  (0.99 to 3.03) | 5  (3 to 9) | 0.14  (0.07 to 0.24) | 96  (48 to 165) | 2.26  (1.13 to 3.83) | 1.18%  (1.08 to 1.28) | 0.88%  (0.79 to 0.96) |
| Bermuda | 1  (1 to 2) | 2.01  (1.16 to 2.99) | 21  (13 to 31) | 35.68  (21.12 to 52.74) | 2  (1 to 3) | 1.35  (0.77 to 2.03) | 34  (20 to 50) | 23.47  (13.8 to 34.75) | -1.33%  (-1.5 to -1.16) | -1.39%  (-1.56 to -1.23) |
| Bhutan | 1  (1 to 2) | 0.6  (0.31 to 0.94) | 38  (19 to 59) | 14.51  (7.29 to 22.57) | 4  (2 to 5) | 0.61  (0.34 to 0.95) | 84  (48 to 128) | 13.59  (7.8 to 20.81) | 0%  (-0.09 to 0.1) | -0.28%  (-0.37 to -0.19) |
| Bolivia (Plurinational State of) | 17  (8 to 29) | 0.68  (0.33 to 1.16) | 355  (166 to 593) | 12.24  (5.76 to 20.34) | 58  (27 to 100) | 0.76  (0.37 to 1.34) | 1111  (536 to 1912) | 13.29  (6.36 to 23.13) | 0.48%  (0.44 to 0.52) | 0.3%  (0.27 to 0.34) |
| Bosnia and Herzegovina | 25  (14 to 38) | 0.71  (0.41 to 1.08) | 540  (316 to 824) | 13.93  (7.93 to 21.26) | 69  (36 to 109) | 1.06  (0.56 to 1.66) | 1276  (677 to 1979) | 19.73  (10.53 to 30.29) | 1.64%  (1.48 to 1.79) | 1.5%  (1.31 to 1.69) |
| Botswana | 4  (2 to 6) | 0.87  (0.49 to 1.34) | 90  (46 to 143) | 17.35  (9.33 to 26.59) | 11  (6 to 17) | 0.93  (0.5 to 1.47) | 246  (131 to 392) | 18.14  (9.89 to 28.68) | 0.43%  (0.21 to 0.65) | 0.31%  (0.08 to 0.54) |
| Brazil | 642  (383 to 890) | 0.89  (0.53 to 1.23) | 13480  (8165 to 18922) | 16.34  (9.74 to 22.72) | 2543  (1514 to 3588) | 1.05  (0.63 to 1.48) | 52158  (31001 to 73625) | 20.89  (12.44 to 29.39) | 0.64%  (0.57 to 0.72) | 0.86%  (0.8 to 0.92) |
| Brunei Darussalam | 2  (1 to 3) | 1.94  (1.08 to 3.02) | 40  (23 to 62) | 39.28  (22.18 to 61.88) | 4  (2 to 6) | 1.46  (0.82 to 2.25) | 96  (57 to 147) | 28.56  (17.09 to 43.31) | -0.19%  (-0.51 to 0.13) | -0.45%  (-0.75 to -0.15) |
| Bulgaria | 101  (57 to 151) | 0.93  (0.51 to 1.39) | 2152  (1231 to 3223) | 17.95  (10.22 to 26.87) | 188  (99 to 294) | 1.23  (0.65 to 1.92) | 3390  (1787 to 5292) | 22.98  (12.43 to 35.81) | 1.42%  (1.19 to 1.65) | 1.22%  (1.05 to 1.4) |
| Burkina Faso | 6  (4 to 10) | 0.18  (0.1 to 0.3) | 140  (78 to 227) | 3.53  (1.97 to 5.76) | 16  (8 to 26) | 0.23  (0.11 to 0.37) | 335  (172 to 547) | 4.09  (2.02 to 6.6) | 0.85%  (0.79 to 0.91) | 0.64%  (0.57 to 0.71) |
| Burundi | 5  (2 to 8) | 0.24  (0.12 to 0.39) | 110  (54 to 181) | 4.97  (2.49 to 8.08) | 7  (4 to 12) | 0.21  (0.11 to 0.36) | 174  (88 to 291) | 3.98  (2.05 to 6.53) | -0.71%  (-0.83 to -0.58) | -1.08%  (-1.24 to -0.93) |
| Cabo Verde | 0  (0 to 1) | 0.12  (0.06 to 0.2) | 5  (3 to 8) | 2.1  (1.13 to 3.3) | 1  (1 to 2) | 0.33  (0.16 to 0.57) | 21  (11 to 35) | 5.15  (2.59 to 8.46) | 2.97%  (2.6 to 3.34) | 2.5%  (2.14 to 2.86) |
| Cambodia | 8  (4 to 13) | 0.25  (0.12 to 0.42) | 171  (75 to 294) | 4.47  (1.97 to 7.58) | 26  (12 to 45) | 0.29  (0.13 to 0.5) | 512  (240 to 869) | 4.93  (2.34 to 8.28) | 0.42%  (0.26 to 0.58) | 0.15%  (-0.02 to 0.33) |
| Cameroon | 10  (6 to 15) | 0.28  (0.16 to 0.45) | 229  (128 to 353) | 5.54  (3.11 to 8.65) | 32  (16 to 53) | 0.35  (0.18 to 0.56) | 735  (370 to 1244) | 6.47  (3.31 to 10.88) | 0.85%  (0.77 to 0.92) | 0.63%  (0.56 to 0.7) |
| Canada | 252  (129 to 407) | 0.78  (0.4 to 1.28) | 4207  (2234 to 6504) | 12.89  (6.82 to 20.02) | 482  (226 to 811) | 0.58  (0.28 to 0.96) | 7070  (3543 to 11521) | 9.07  (4.64 to 14.77) | -0.73%  (-0.84 to -0.62) | -0.86%  (-0.97 to -0.76) |
| Central African Republic | 3  (1 to 4) | 0.29  (0.15 to 0.48) | 70  (34 to 115) | 6.35  (3.25 to 10.2) | 5  (2 to 8) | 0.29  (0.14 to 0.5) | 132  (63 to 241) | 6.1  (2.99 to 10.96) | -0.09%  (-0.15 to -0.04) | -0.18%  (-0.24 to -0.13) |
| Chad | 4  (2 to 6) | 0.16  (0.08 to 0.24) | 84  (46 to 131) | 3.11  (1.68 to 4.79) | 10  (5 to 17) | 0.23  (0.12 to 0.37) | 240  (127 to 392) | 4.48  (2.37 to 7.29) | 1.54%  (1.41 to 1.67) | 1.45%  (1.34 to 1.56) |
| Chile | 48  (27 to 79) | 0.54  (0.29 to 0.89) | 943  (549 to 1522) | 9.83  (5.65 to 15.76) | 149  (80 to 238) | 0.57  (0.3 to 0.9) | 2707  (1543 to 4217) | 10.43  (5.95 to 16.2) | 0.43%  (0.31 to 0.55) | 0.46%  (0.35 to 0.58) |
| China | 5735  (3369 to 8371) | 0.93  (0.55 to 1.36) | 128353  (74847 to 188832) | 17.12  (10.06 to 24.93) | 16698  (10065 to 24626) | 0.87  (0.53 to 1.29) | 320464  (192275 to 474070) | 15.63  (9.47 to 22.88) | -0.33%  (-0.41 to -0.25) | -0.4%  (-0.52 to -0.28) |
| Colombia | 100  (54 to 142) | 0.63  (0.34 to 0.9) | 2423  (1365 to 3402) | 13.67  (7.51 to 19.16) | 429  (243 to 640) | 0.78  (0.44 to 1.16) | 10243  (5876 to 15088) | 18.55  (10.67 to 27.28) | 0.69%  (0.45 to 0.93) | 1.11%  (0.79 to 1.43) |
| Comoros | 0  (0 to 1) | 0.21  (0.11 to 0.35) | 8  (4 to 14) | 4.41  (2.21 to 7.37) | 1  (1 to 2) | 0.25  (0.13 to 0.44) | 23  (12 to 41) | 4.98  (2.48 to 8.48) | 0.54%  (0.44 to 0.64) | 0.37%  (0.23 to 0.51) |
| Congo | 3  (1 to 5) | 0.33  (0.17 to 0.52) | 76  (37 to 122) | 7.18  (3.61 to 11.4) | 7  (4 to 11) | 0.31  (0.16 to 0.51) | 183  (96 to 292) | 6.7  (3.59 to 10.54) | -0.2%  (-0.36 to -0.04) | -0.33%  (-0.53 to -0.13) |
| Cook Islands | 0  (0 to 0) | 0.47  (0.26 to 0.73) | 1  (1 to 2) | 10.24  (5.69 to 15.85) | 0  (0 to 0) | 0.34  (0.19 to 0.54) | 2  (1 to 3) | 7.36  (4.11 to 11.43) | -1.15%  (-1.32 to -0.98) | -1.18%  (-1.37 to -0.99) |
| Costa Rica | 7  (4 to 11) | 0.43  (0.23 to 0.69) | 137  (77 to 212) | 8.08  (4.55 to 12.55) | 37  (20 to 58) | 0.67  (0.37 to 1.05) | 710  (402 to 1085) | 12.95  (7.32 to 19.69) | 1.64%  (1.47 to 1.82) | 1.75%  (1.58 to 1.93) |
| Côte d'Ivoire | 5  (3 to 7) | 0.16  (0.09 to 0.25) | 119  (62 to 186) | 3.29  (1.76 to 5.04) | 14  (8 to 24) | 0.17  (0.09 to 0.29) | 349  (185 to 602) | 3.38  (1.84 to 5.71) | 0.18%  (0.1 to 0.25) | 0.14%  (0.08 to 0.21) |
| Croatia | 85  (50 to 122) | 1.62  (0.96 to 2.33) | 1494  (892 to 2118) | 26.85  (16.18 to 37.91) | 164  (95 to 249) | 1.6  (0.94 to 2.38) | 2602  (1521 to 3875) | 26.7  (15.81 to 39.5) | 0.24%  (0.06 to 0.42) | 0.25%  (0.08 to 0.43) |
| Cuba | 107  (59 to 160) | 1.08  (0.58 to 1.61) | 2056  (1169 to 2988) | 20.18  (11.4 to 29.31) | 260  (146 to 393) | 1.25  (0.72 to 1.89) | 4636  (2662 to 7043) | 23.14  (13.2 to 35.31) | 0.54%  (0.45 to 0.62) | 0.54%  (0.43 to 0.64) |
| Cyprus | 10  (6 to 15) | 1.71  (0.93 to 2.6) | 180  (101 to 269) | 26.37  (14.72 to 39.33) | 21  (11 to 32) | 1.09  (0.6 to 1.67) | 351  (200 to 528) | 17.37  (9.88 to 26.16) | -1.09%  (-1.29 to -0.89) | -1.01%  (-1.17 to -0.86) |
| Czechia | 317  (179 to 456) | 2.25  (1.27 to 3.24) | 5731  (3251 to 8080) | 40.29  (22.57 to 56.87) | 353  (215 to 523) | 1.48  (0.9 to 2.19) | 6086  (3760 to 9124) | 26.4  (16.4 to 39.72) | -1.6%  (-1.77 to -1.43) | -1.58%  (-1.71 to -1.44) |
| Democratic People's Republic of Korea | 82  (44 to 141) | 0.65  (0.36 to 1.11) | 1932  (1020 to 3339) | 12.85  (6.9 to 22.08) | 173  (89 to 298) | 0.56  (0.29 to 0.96) | 3719  (1903 to 6612) | 11.42  (5.81 to 20.09) | -0.29%  (-0.41 to -0.17) | -0.25%  (-0.35 to -0.14) |
| Democratic Republic of the Congo | 41  (23 to 64) | 0.38  (0.22 to 0.59) | 964  (545 to 1500) | 7.23  (4.09 to 11.07) | 97  (52 to 164) | 0.38  (0.2 to 0.67) | 2213  (1166 to 3691) | 7.14  (3.84 to 11.98) | 0.02%  (-0.19 to 0.23) | 0%  (-0.23 to 0.22) |
| Denmark | 152  (93 to 219) | 1.71  (1.04 to 2.45) | 2612  (1588 to 3768) | 30.75  (18.51 to 44.32) | 201  (116 to 307) | 1.46  (0.85 to 2.22) | 3114  (1801 to 4659) | 23.98  (13.9 to 35.66) | -0.92%  (-1.22 to -0.61) | -1.19%  (-1.46 to -0.92) |
| Djibouti | 0  (0 to 0) | 0.24  (0.13 to 0.42) | 6  (3 to 11) | 4.83  (2.51 to 8.43) | 1  (1 to 3) | 0.34  (0.17 to 0.59) | 35  (18 to 64) | 6.3  (3.21 to 10.82) | 1.11%  (1.05 to 1.17) | 0.88%  (0.82 to 0.94) |
| Dominica | 0  (0 to 1) | 0.73  (0.38 to 1.12) | 7  (4 to 11) | 12.28  (6.59 to 18.37) | 1  (0 to 1) | 0.84  (0.47 to 1.36) | 11  (6 to 17) | 13.61  (7.66 to 21.84) | 0.58%  (0.47 to 0.69) | 0.44%  (0.35 to 0.52) |
| Dominican Republic | 15  (9 to 22) | 0.52  (0.29 to 0.78) | 317  (187 to 464) | 9.25  (5.39 to 13.4) | 56  (31 to 92) | 0.58  (0.32 to 0.96) | 1118  (633 to 1765) | 11.29  (6.37 to 17.92) | 0.94%  (0.65 to 1.23) | 1.1%  (0.91 to 1.29) |
| Ecuador | 11  (6 to 18) | 0.24  (0.12 to 0.41) | 226  (117 to 340) | 4.5  (2.37 to 6.88) | 48  (25 to 84) | 0.31  (0.16 to 0.54) | 993  (513 to 1651) | 6.13  (3.15 to 10.18) | 1.32%  (1.01 to 1.64) | 1.38%  (1.1 to 1.66) |
| Egypt | 96  (57 to 139) | 0.42  (0.25 to 0.62) | 2752  (1627 to 3957) | 9.61  (5.68 to 13.75) | 325  (186 to 483) | 0.65  (0.37 to 0.98) | 8584  (4889 to 12999) | 13.76  (7.93 to 20.58) | 2.16%  (1.84 to 2.47) | 1.73%  (1.47 to 1.98) |
| El Salvador | 6  (3 to 9) | 0.22  (0.11 to 0.32) | 124  (67 to 183) | 4.24  (2.28 to 6.28) | 23  (11 to 37) | 0.35  (0.18 to 0.56) | 438  (227 to 692) | 7.02  (3.67 to 11.07) | 1.69%  (1.52 to 1.86) | 1.7%  (1.54 to 1.86) |
| Equatorial Guinea | 0  (0 to 1) | 0.31  (0.17 to 0.49) | 11  (6 to 18) | 6.26  (3.48 to 9.86) | 2  (1 to 3) | 0.45  (0.22 to 0.79) | 39  (20 to 68) | 8.44  (4.25 to 14.86) | 1.59%  (1.47 to 1.71) | 1.28%  (1.16 to 1.4) |
| Eritrea | 1  (0 to 2) | 0.12  (0.06 to 0.2) | 25  (12 to 47) | 2.44  (1.21 to 4.18) | 3  (1 to 5) | 0.15  (0.07 to 0.27) | 73  (36 to 125) | 2.94  (1.47 to 4.99) | 0.98%  (0.93 to 1.04) | 0.74%  (0.7 to 0.79) |
| Estonia | 19  (11 to 28) | 0.93  (0.54 to 1.36) | 346  (205 to 512) | 16.52  (9.76 to 24.21) | 36  (19 to 56) | 1.07  (0.59 to 1.65) | 541  (308 to 828) | 17.46  (9.92 to 26.65) | 0.27%  (0.14 to 0.4) | -0.01%  (-0.15 to 0.13) |
| Eswatini | 1  (1 to 2) | 0.66  (0.34 to 1.04) | 35  (18 to 55) | 13.09  (6.94 to 20.44) | 4  (2 to 6) | 0.87  (0.44 to 1.43) | 96  (46 to 154) | 17.89  (9.09 to 28.87) | 1.28%  (0.86 to 1.7) | 1.36%  (0.9 to 1.84) |
| Ethiopia | 78  (33 to 127) | 0.51  (0.22 to 0.83) | 2015  (835 to 3297) | 10.74  (4.63 to 17.42) | 151  (80 to 241) | 0.43  (0.22 to 0.7) | 3323  (1772 to 5304) | 8.19  (4.37 to 13.1) | -0.81%  (-1.03 to -0.58) | -1.14%  (-1.39 to -0.89) |
| Fiji | 2  (1 to 2) | 0.5  (0.29 to 0.76) | 45  (26 to 69) | 11.74  (6.72 to 17.94) | 4  (2 to 6) | 0.57  (0.31 to 0.9) | 97  (52 to 154) | 12.47  (6.87 to 19.32) | 0.41%  (0.25 to 0.57) | 0.23%  (0.05 to 0.42) |
| Finland | 88  (51 to 126) | 1.18  (0.69 to 1.69) | 1525  (898 to 2154) | 20.67  (12.23 to 29.38) | 134  (74 to 197) | 0.87  (0.5 to 1.28) | 2103  (1247 to 3131) | 15.02  (8.88 to 22.61) | -1.04%  (-1.19 to -0.89) | -1.08%  (-1.24 to -0.93) |
| France | 1932  (1149 to 2862) | 2.12  (1.26 to 3.1) | 31447  (18717 to 44607) | 36.12  (21.47 to 51.82) | 2368  (1355 to 3527) | 1.3  (0.77 to 1.89) | 35039  (21059 to 50657) | 22.44  (13.57 to 32.46) | -1.61%  (-1.69 to -1.53) | -1.54%  (-1.6 to -1.48) |
| Gabon | 1  (1 to 2) | 0.26  (0.11 to 0.48) | 26  (11 to 46) | 4.84  (2.12 to 8.78) | 2  (1 to 4) | 0.28  (0.13 to 0.5) | 44  (22 to 77) | 4.85  (2.4 to 8.46) | 0.04%  (-0.06 to 0.14) | -0.15%  (-0.26 to -0.04) |
| Gambia | 0  (0 to 0) | 0.09  (0.05 to 0.15) | 6  (3 to 9) | 1.71  (0.88 to 2.77) | 1  (0 to 1) | 0.11  (0.06 to 0.18) | 19  (10 to 32) | 2.06  (1.08 to 3.44) | 0.55%  (0.45 to 0.64) | 0.48%  (0.34 to 0.62) |
| Georgia | 27  (15 to 38) | 0.45  (0.25 to 0.65) | 546  (293 to 805) | 8.89  (4.83 to 12.96) | 43  (24 to 64) | 0.68  (0.38 to 1.01) | 771  (434 to 1163) | 12.6  (6.94 to 18.92) | 2.78%  (2.09 to 3.48) | 2.45%  (1.83 to 3.06) |
| Germany | 2503  (1432 to 3708) | 1.81  (1.03 to 2.69) | 41055  (24293 to 60281) | 30.45  (17.95 to 44.18) | 2168  (1186 to 3373) | 0.9  (0.49 to 1.38) | 32876  (18078 to 50276) | 15.21  (8.16 to 23.19) | -2.67%  (-2.84 to -2.51) | -2.63%  (-2.81 to -2.45) |
| Ghana | 13  (7 to 20) | 0.27  (0.15 to 0.43) | 299  (158 to 472) | 5.22  (2.88 to 8.3) | 44  (24 to 67) | 0.36  (0.2 to 0.57) | 978  (537 to 1471) | 6.56  (3.59 to 9.91) | 1.12%  (0.95 to 1.28) | 0.88%  (0.61 to 1.16) |
| Greece | 162  (92 to 241) | 1.09  (0.62 to 1.62) | 2673  (1578 to 3923) | 17.4  (10.25 to 25.57) | 293  (159 to 454) | 0.9  (0.51 to 1.37) | 4162  (2374 to 6305) | 14.69  (8.57 to 21.91) | -1.08%  (-1.35 to -0.81) | -0.98%  (-1.21 to -0.75) |
| Greenland | 1  (0 to 1) | 2.34  (1.31 to 3.78) | 12  (6 to 19) | 42.28  (23.05 to 66.6) | 1  (0 to 1) | 1.29  (0.71 to 2.02) | 14  (8 to 23) | 22.62  (12.43 to 34.5) | -2.03%  (-2.15 to -1.92) | -2.12%  (-2.23 to -2) |
| Grenada | 1  (0 to 1) | 0.81  (0.44 to 1.22) | 12  (6 to 17) | 15.63  (8.39 to 23.57) | 1  (1 to 2) | 1.11  (0.64 to 1.65) | 21  (12 to 31) | 19.76  (11.1 to 29.65) | 1.51%  (1.31 to 1.71) | 1.13%  (0.99 to 1.27) |
| Guam | 0  (0 to 1) | 0.68  (0.37 to 1.08) | 8  (4 to 12) | 11.93  (6.85 to 17.96) | 1  (0 to 1) | 0.33  (0.19 to 0.5) | 16  (9 to 23) | 7.5  (4.25 to 11.07) | -1.6%  (-2.03 to -1.17) | -1%  (-1.39 to -0.6) |
| Guatemala | 3  (2 to 5) | 0.14  (0.07 to 0.24) | 60  (31 to 98) | 2.2  (1.15 to 3.63) | 18  (9 to 30) | 0.19  (0.09 to 0.32) | 334  (180 to 534) | 3.23  (1.7 to 5.26) | 0.69%  (0.33 to 1.05) | 1.09%  (0.82 to 1.35) |
| Guinea | 3  (1 to 4) | 0.09  (0.05 to 0.14) | 54  (28 to 85) | 1.71  (0.89 to 2.73) | 5  (2 to 8) | 0.1  (0.05 to 0.18) | 97  (51 to 166) | 1.86  (0.99 to 3.18) | 0.43%  (0.37 to 0.5) | 0.27%  (0.23 to 0.32) |
| Guinea-Bissau | 1  (0 to 2) | 0.3  (0.16 to 0.48) | 23  (12 to 38) | 6.2  (3.23 to 10.16) | 2  (1 to 3) | 0.39  (0.21 to 0.63) | 50  (27 to 80) | 7.55  (4.08 to 12.24) | 1.14%  (1.06 to 1.23) | 0.84%  (0.77 to 0.92) |
| Guyana | 2  (1 to 3) | 0.7  (0.4 to 1.02) | 49  (28 to 72) | 13.67  (7.82 to 20.09) | 5  (2 to 7) | 0.84  (0.44 to 1.3) | 102  (53 to 163) | 16.55  (8.81 to 25.73) | 0.84%  (0.56 to 1.12) | 0.9%  (0.65 to 1.15) |
| Haiti | 24  (12 to 40) | 0.96  (0.49 to 1.61) | 565  (268 to 962) | 18.98  (9.35 to 31.75) | 51  (25 to 86) | 0.91  (0.45 to 1.55) | 1202  (558 to 2096) | 17.92  (8.71 to 30.64) | -0.07%  (-0.14 to 0) | -0.03%  (-0.11 to 0.05) |
| Honduras | 3  (2 to 5) | 0.17  (0.09 to 0.27) | 69  (36 to 111) | 3.43  (1.78 to 5.47) | 15  (8 to 25) | 0.28  (0.14 to 0.47) | 336  (177 to 560) | 5.5  (2.88 to 9.09) | 1.7%  (1.46 to 1.94) | 1.63%  (1.43 to 1.83) |
| Hungary | 271  (157 to 409) | 1.88  (1.07 to 2.84) | 4821  (2820 to 7163) | 32.36  (18.63 to 47.91) | 333  (183 to 502) | 1.53  (0.85 to 2.31) | 5813  (3190 to 8859) | 28.2  (15.61 to 43.18) | -0.76%  (-1.02 to -0.5) | -0.54%  (-0.77 to -0.31) |
| Iceland | 4  (2 to 5) | 1.17  (0.7 to 1.69) | 63  (38 to 90) | 21.37  (12.84 to 30.73) | 6  (3 to 9) | 0.89  (0.53 to 1.34) | 96  (56 to 141) | 15.64  (9.3 to 23.23) | -0.74%  (-0.88 to -0.6) | -0.91%  (-1.02 to -0.79) |
| India | 1004  (604 to 1422) | 0.27  (0.16 to 0.38) | 24769  (14823 to 35024) | 5.63  (3.39 to 8) | 3005  (1863 to 4279) | 0.29  (0.18 to 0.41) | 64281  (39426 to 91649) | 5.64  (3.48 to 8.04) | 0.08%  (-0.07 to 0.23) | -0.28%  (-0.43 to -0.13) |
| Indonesia | 976  (567 to 1452) | 1.22  (0.71 to 1.82) | 23714  (13845 to 35511) | 25.46  (14.77 to 37.91) | 3195  (1875 to 4703) | 1.7  (1 to 2.49) | 73564  (42224 to 110118) | 32.95  (19.23 to 48.48) | 1.06%  (0.97 to 1.16) | 0.8%  (0.71 to 0.88) |
| Iran (Islamic Republic of) | 115  (67 to 172) | 0.55  (0.33 to 0.83) | 3065  (1801 to 4518) | 11.87  (6.98 to 17.74) | 401  (236 to 564) | 0.57  (0.34 to 0.81) | 9322  (5441 to 13028) | 12  (6.99 to 16.7) | 0.55%  (0.32 to 0.78) | 0.52%  (0.27 to 0.76) |
| Iraq | 37  (23 to 54) | 0.48  (0.29 to 0.7) | 1011  (594 to 1489) | 12.03  (7.13 to 17.69) | 126  (71 to 192) | 0.6  (0.34 to 0.89) | 3426  (1915 to 5203) | 13.59  (7.58 to 20.57) | 0.77%  (0.55 to 0.99) | 0.45%  (0.28 to 0.62) |
| Ireland | 97  (60 to 140) | 2.39  (1.46 to 3.44) | 1752  (1074 to 2479) | 42.45  (26.1 to 59.71) | 116  (67 to 176) | 1.37  (0.79 to 2.06) | 1960  (1140 to 2903) | 23.82  (13.88 to 34.86) | -1.62%  (-1.7 to -1.53) | -1.74%  (-1.83 to -1.66) |
| Israel | 74  (43 to 109) | 1.58  (0.91 to 2.33) | 1320  (755 to 1941) | 27.23  (15.68 to 40.03) | 143  (77 to 221) | 1.03  (0.57 to 1.58) | 2194  (1230 to 3351) | 16.83  (9.56 to 25.3) | -2.02%  (-2.34 to -1.69) | -2.14%  (-2.46 to -1.82) |
| Italy | 1619  (981 to 2310) | 1.77  (1.07 to 2.51) | 29553  (17672 to 41695) | 32.64  (19.55 to 45.74) | 2316  (1422 to 3386) | 1.27  (0.78 to 1.84) | 36075  (21893 to 51638) | 22.8  (13.8 to 32.2) | -1.13%  (-1.25 to -1.02) | -1.25%  (-1.36 to -1.14) |
| Jamaica | 19  (11 to 29) | 1.04  (0.61 to 1.53) | 368  (225 to 529) | 20.24  (12.48 to 29.16) | 43  (25 to 65) | 1.38  (0.8 to 2.08) | 876  (514 to 1309) | 28.47  (16.73 to 42.6) | 0.89%  (0.62 to 1.15) | 1.11%  (0.81 to 1.41) |
| Japan | 2432  (1424 to 3466) | 1.49  (0.87 to 2.15) | 54728  (32149 to 76358) | 32.4  (18.93 to 45.18) | 5747  (3143 to 8841) | 1.26  (0.74 to 1.84) | 93063  (54354 to 135399) | 26.59  (15.64 to 37.11) | -0.59%  (-0.65 to -0.53) | -0.71%  (-0.77 to -0.65) |
| Jordan | 9  (5 to 14) | 0.82  (0.47 to 1.27) | 237  (135 to 376) | 17.8  (10.32 to 27.52) | 42  (23 to 66) | 0.71  (0.39 to 1.14) | 1006  (553 to 1610) | 14.08  (7.87 to 22.2) | -0.33%  (-0.55 to -0.12) | -0.7%  (-0.94 to -0.46) |
| Kazakhstan | 79  (43 to 119) | 0.67  (0.36 to 1.03) | 1836  (1012 to 2751) | 14.64  (8.07 to 22.14) | 80  (46 to 124) | 0.5  (0.29 to 0.77) | 1825  (968 to 2791) | 10.3  (5.69 to 15.63) | -0.56%  (-0.86 to -0.27) | -0.81%  (-1.05 to -0.57) |
| Kenya | 15  (8 to 23) | 0.2  (0.11 to 0.3) | 400  (219 to 615) | 4.63  (2.61 to 7.12) | 61  (34 to 99) | 0.31  (0.17 to 0.5) | 1610  (891 to 2514) | 6.82  (3.75 to 11.01) | 2.11%  (1.86 to 2.36) | 1.76%  (1.55 to 1.98) |
| Kiribati | 0  (0 to 0) | 0.74  (0.44 to 1.15) | 7  (4 to 11) | 18.21  (10.74 to 27.76) | 0  (0 to 1) | 0.76  (0.41 to 1.19) | 14  (8 to 23) | 18.07  (10 to 28.18) | -0.04%  (-0.15 to 0.07) | -0.14%  (-0.24 to -0.04) |
| Kuwait | 4  (2 to 5) | 0.74  (0.45 to 1.05) | 109  (68 to 153) | 16.49  (10.11 to 23.06) | 26  (15 to 37) | 1  (0.59 to 1.45) | 720  (427 to 1040) | 21.68  (12.88 to 31.27) | 1.47%  (0.99 to 1.95) | 1.26%  (0.74 to 1.79) |
| Kyrgyzstan | 13  (7 to 19) | 0.47  (0.27 to 0.7) | 271  (155 to 398) | 9.47  (5.43 to 13.91) | 12  (7 to 19) | 0.33  (0.18 to 0.52) | 263  (148 to 400) | 6.16  (3.45 to 9.54) | -0.8%  (-1.12 to -0.47) | -1.08%  (-1.33 to -0.83) |
| Lao People's Democratic Republic | 5  (2 to 9) | 0.32  (0.15 to 0.55) | 131  (56 to 227) | 6.64  (2.96 to 11.49) | 12  (6 to 19) | 0.32  (0.16 to 0.54) | 260  (128 to 436) | 6.12  (3.13 to 10.11) | -0.18%  (-0.27 to -0.09) | -0.48%  (-0.6 to -0.36) |
| Latvia | 31  (18 to 46) | 0.87  (0.5 to 1.27) | 652  (375 to 959) | 18.04  (10.38 to 26.61) | 40  (22 to 61) | 0.87  (0.5 to 1.33) | 700  (403 to 1062) | 16.88  (9.68 to 25.52) | 0.14%  (-0.05 to 0.34) | -0.14%  (-0.31 to 0.03) |
| Lebanon | 21  (11 to 32) | 1.12  (0.59 to 1.73) | 479  (252 to 745) | 22.85  (12.11 to 34.94) | 61  (34 to 95) | 0.94  (0.52 to 1.48) | 1115  (631 to 1738) | 18.03  (10.21 to 28.2) | -0.13%  (-0.35 to 0.1) | -0.32%  (-0.53 to -0.11) |
| Lesotho | 2  (1 to 3) | 0.26  (0.13 to 0.42) | 28  (15 to 45) | 3.96  (2.09 to 6.28) | 3  (2 to 5) | 0.46  (0.22 to 0.81) | 60  (30 to 101) | 7.26  (3.57 to 12.69) | 2.64%  (2.19 to 3.09) | 2.68%  (2.24 to 3.12) |
| Liberia | 3  (2 to 4) | 0.28  (0.16 to 0.43) | 68  (38 to 105) | 6.02  (3.47 to 9.13) | 6  (3 to 10) | 0.35  (0.17 to 0.6) | 157  (75 to 275) | 7.22  (3.59 to 12.51) | 1.05%  (0.85 to 1.25) | 0.93%  (0.73 to 1.12) |
| Libya | 21  (12 to 32) | 1.22  (0.7 to 1.9) | 476  (281 to 726) | 25.55  (15.02 to 39.11) | 57  (31 to 90) | 1.26  (0.69 to 2) | 1387  (761 to 2204) | 26.3  (14.37 to 41.64) | 0.6%  (0.39 to 0.81) | 0.49%  (0.31 to 0.67) |
| Lithuania | 48  (29 to 73) | 1.06  (0.63 to 1.61) | 920  (533 to 1369) | 20.28  (11.66 to 30.26) | 86  (50 to 128) | 1.27  (0.74 to 1.89) | 1432  (818 to 2136) | 23.1  (13.12 to 34.46) | 0.65%  (0.5 to 0.8) | 0.46%  (0.32 to 0.61) |
| Luxembourg | 11  (6 to 16) | 2.03  (1.14 to 2.94) | 192  (112 to 275) | 34.73  (20.05 to 49.23) | 14  (8 to 21) | 1.13  (0.66 to 1.72) | 208  (121 to 307) | 18.34  (10.49 to 27.07) | -1.91%  (-2.07 to -1.75) | -2.07%  (-2.22 to -1.92) |
| Madagascar | 8  (4 to 14) | 0.2  (0.1 to 0.33) | 202  (102 to 334) | 4.14  (2.05 to 6.97) | 16  (9 to 27) | 0.21  (0.11 to 0.35) | 417  (224 to 708) | 4.08  (2.2 to 6.61) | 0.22%  (0.12 to 0.32) | -0.02%  (-0.12 to 0.08) |
| Malawi | 3  (2 to 5) | 0.09  (0.05 to 0.14) | 67  (36 to 114) | 1.82  (1.01 to 3.01) | 6  (3 to 10) | 0.1  (0.05 to 0.17) | 143  (75 to 237) | 2.06  (1.09 to 3.43) | 0.33%  (0.2 to 0.46) | 0.25%  (0.12 to 0.39) |
| Malaysia | 90  (51 to 133) | 1.09  (0.62 to 1.6) | 2098  (1176 to 3076) | 23.3  (13.08 to 34.25) | 340  (202 to 503) | 1.34  (0.77 to 1.98) | 7763  (4559 to 11443) | 27.72  (16.22 to 41.12) | 0.53%  (0.4 to 0.67) | 0.47%  (0.34 to 0.6) |
| Maldives | 1  (0 to 1) | 1.04  (0.59 to 1.51) | 23  (11 to 35) | 24.51  (12.82 to 35.97) | 2  (1 to 3) | 0.63  (0.38 to 0.92) | 42  (25 to 62) | 12.59  (7.47 to 18.45) | -1.96%  (-2.08 to -1.84) | -2.54%  (-2.68 to -2.4) |
| Mali | 10  (6 to 16) | 0.31  (0.18 to 0.49) | 251  (146 to 392) | 6.54  (3.87 to 10.14) | 24  (13 to 39) | 0.33  (0.18 to 0.53) | 566  (315 to 937) | 6.71  (3.8 to 11.03) | 0.36%  (0.27 to 0.46) | 0.28%  (0.17 to 0.39) |
| Malta | 7  (4 to 10) | 1.78  (1.1 to 2.52) | 141  (86 to 201) | 33.26  (20.33 to 47.35) | 15  (9 to 22) | 1.37  (0.81 to 2.01) | 257  (153 to 374) | 25.26  (15.05 to 36.62) | -0.99%  (-1.11 to -0.87) | -1.03%  (-1.14 to -0.92) |
| Marshall Islands | 0  (0 to 0) | 1.48  (0.93 to 2.14) | 6  (4 to 9) | 36.93  (22.41 to 53.61) | 0  (0 to 1) | 1.48  (0.89 to 2.26) | 15  (8 to 24) | 37.7  (21.86 to 57.55) | -0.06%  (-0.13 to 0.02) | 0.01%  (-0.08 to 0.1) |
| Mauritania | 5  (3 to 7) | 0.52  (0.28 to 0.81) | 112  (62 to 172) | 11.39  (6.23 to 17.54) | 11  (6 to 18) | 0.59  (0.32 to 0.92) | 261  (140 to 407) | 12.29  (6.65 to 19.18) | 0.47%  (0.31 to 0.63) | 0.3%  (0.14 to 0.46) |
| Mauritius | 2  (1 to 4) | 0.41  (0.24 to 0.62) | 54  (32 to 79) | 7.9  (4.8 to 11.59) | 12  (7 to 18) | 0.67  (0.37 to 1.02) | 242  (143 to 367) | 13.12  (7.83 to 19.96) | 1.12%  (0.94 to 1.31) | 1.14%  (0.95 to 1.33) |
| Mexico | 113  (62 to 163) | 0.33  (0.18 to 0.48) | 2203  (1248 to 3155) | 5.66  (3.17 to 8.1) | 465  (278 to 661) | 0.39  (0.23 to 0.56) | 10054  (5945 to 14432) | 8.01  (4.77 to 11.4) | 0.59%  (0.17 to 1) | 1.14%  (0.71 to 1.58) |
| Micronesia (Federated States of) | 1  (0 to 1) | 1.71  (1 to 2.57) | 21  (12 to 32) | 41.71  (24.1 to 63.7) | 1  (1 to 2) | 1.65  (0.93 to 2.49) | 32  (18 to 49) | 39.6  (22.49 to 60.82) | -0.15%  (-0.17 to -0.12) | -0.2%  (-0.22 to -0.17) |
| Monaco | 1  (1 to 2) | 1.8  (0.94 to 2.8) | 24  (13 to 38) | 32.33  (17.92 to 50.25) | 2  (1 to 4) | 1.95  (1.11 to 2.97) | 36  (21 to 55) | 34.52  (20.04 to 52.54) | 0.33%  (0.23 to 0.42) | 0.28%  (0.18 to 0.38) |
| Mongolia | 2  (1 to 3) | 0.18  (0.1 to 0.29) | 40  (20 to 67) | 3.88  (1.96 to 6.39) | 4  (2 to 6) | 0.21  (0.11 to 0.34) | 87  (46 to 139) | 4.12  (2.21 to 6.59) | 0.25%  (0.08 to 0.41) | -0.07%  (-0.25 to 0.12) |
| Montenegro | 4  (2 to 6) | 0.74  (0.42 to 1.11) | 84  (47 to 123) | 13.83  (7.93 to 20.46) | 10  (6 to 15) | 1.09  (0.6 to 1.66) | 178  (101 to 273) | 18.44  (10.43 to 28.07) | 1.41%  (1.2 to 1.61) | 1.06%  (0.95 to 1.17) |
| Morocco | 69  (39 to 106) | 0.53  (0.3 to 0.8) | 1678  (928 to 2643) | 11.69  (6.5 to 18.34) | 237  (128 to 359) | 0.76  (0.41 to 1.15) | 5614  (2986 to 8624) | 16.29  (8.79 to 24.85) | 1.42%  (1.31 to 1.52) | 1.3%  (1.2 to 1.39) |
| Mozambique | 4  (2 to 6) | 0.09  (0.05 to 0.15) | 79  (43 to 129) | 1.61  (0.85 to 2.58) | 9  (4 to 15) | 0.12  (0.06 to 0.22) | 185  (94 to 312) | 2.09  (1.04 to 3.52) | 1.39%  (1.23 to 1.55) | 1.29%  (1.14 to 1.45) |
| Myanmar | 33  (15 to 61) | 0.17  (0.08 to 0.31) | 768  (337 to 1417) | 3.5  (1.63 to 6.43) | 83  (42 to 142) | 0.2  (0.1 to 0.33) | 1796  (903 to 3097) | 3.87  (1.97 to 6.57) | 0.44%  (0.34 to 0.54) | 0.29%  (0.13 to 0.46) |
| Namibia | 2  (1 to 3) | 0.34  (0.2 to 0.52) | 45  (26 to 66) | 7.13  (4.08 to 10.58) | 5  (2 to 7) | 0.41  (0.21 to 0.61) | 114  (63 to 176) | 8.46  (4.55 to 12.91) | 0.52%  (0.34 to 0.71) | 0.48%  (0.27 to 0.68) |
| Nauru | 0  (0 to 0) | 0.95  (0.46 to 1.62) | 1  (1 to 2) | 23.31  (11.24 to 39.72) | 0  (0 to 0) | 0.97  (0.48 to 1.59) | 1  (1 to 2) | 22.88  (11.21 to 37.95) | 0%  (-0.03 to 0.04) | -0.12%  (-0.16 to -0.08) |
| Nepal | 11  (5 to 18) | 0.14  (0.07 to 0.23) | 270  (126 to 449) | 3  (1.43 to 4.96) | 32  (17 to 51) | 0.16  (0.08 to 0.26) | 713  (373 to 1167) | 3.17  (1.72 to 5.17) | 0.46%  (0.09 to 0.83) | 0.3%  (-0.09 to 0.69) |
| Netherlands | 283  (154 to 441) | 1.35  (0.74 to 2.09) | 4891  (2757 to 7373) | 23.7  (13.27 to 36.01) | 424  (227 to 679) | 1.07  (0.58 to 1.69) | 7071  (3802 to 11159) | 18.72  (10.09 to 29.11) | -0.74%  (-0.87 to -0.62) | -0.74%  (-0.87 to -0.62) |
| New Zealand | 91  (54 to 130) | 2.32  (1.4 to 3.33) | 1601  (940 to 2330) | 40.26  (23.65 to 58.37) | 164  (95 to 247) | 1.77  (1.02 to 2.65) | 2639  (1552 to 3819) | 29.84  (17.61 to 42.92) | -0.64%  (-0.77 to -0.52) | -0.72%  (-0.84 to -0.59) |
| Nicaragua | 3  (1 to 4) | 0.19  (0.1 to 0.31) | 54  (30 to 85) | 3.69  (2.04 to 5.81) | 11  (6 to 18) | 0.25  (0.13 to 0.42) | 233  (124 to 395) | 4.91  (2.57 to 8.21) | 1.32%  (1.03 to 1.62) | 1.35%  (1.09 to 1.62) |
| Niger | 4  (2 to 6) | 0.2  (0.1 to 0.31) | 97  (52 to 155) | 3.83  (2.07 to 6.02) | 14  (8 to 23) | 0.23  (0.13 to 0.38) | 322  (172 to 537) | 4.37  (2.39 to 7.03) | 0.84%  (0.73 to 0.94) | 0.61%  (0.53 to 0.7) |
| Nigeria | 97  (57 to 147) | 0.26  (0.15 to 0.4) | 2172  (1245 to 3260) | 5.19  (3.07 to 7.74) | 219  (117 to 324) | 0.31  (0.17 to 0.45) | 4977  (2673 to 7585) | 5.84  (3.17 to 8.63) | 0.66%  (0.57 to 0.74) | 0.55%  (0.46 to 0.63) |
| Niue | 0  (0 to 0) | 0.67  (0.39 to 1.04) | 0  (0 to 0) | 14.78  (8.48 to 22.35) | 0  (0 to 0) | 0.68  (0.37 to 1.07) | 0  (0 to 0) | 14.71  (8.25 to 22.37) | -0.07%  (-0.1 to -0.03) | -0.18%  (-0.22 to -0.13) |
| North Macedonia | 14  (7 to 20) | 0.84  (0.46 to 1.26) | 281  (152 to 413) | 15.96  (8.61 to 23.07) | 33  (17 to 51) | 1.19  (0.64 to 1.89) | 620  (340 to 954) | 19.96  (10.86 to 31.06) | 1.11%  (0.7 to 1.52) | 0.73%  (0.4 to 1.06) |
| Northern Mariana Islands | 0  (0 to 0) | 0.85  (0.47 to 1.36) | 4  (2 to 6) | 18.72  (10.43 to 29.47) | 0  (0 to 1) | 0.83  (0.44 to 1.25) | 10  (5 to 14) | 17.97  (10.01 to 26.77) | -0.18%  (-0.42 to 0.07) | -0.2%  (-0.45 to 0.06) |
| Norway | 137  (76 to 204) | 1.79  (0.99 to 2.67) | 2319  (1298 to 3353) | 32.26  (18.18 to 46.17) | 172  (97 to 257) | 1.47  (0.84 to 2.18) | 2672  (1580 to 3824) | 24.71  (14.55 to 35.43) | -0.68%  (-0.76 to -0.59) | -0.88%  (-0.96 to -0.8) |
| Oman | 2  (1 to 3) | 0.36  (0.21 to 0.56) | 50  (28 to 80) | 7.62  (4.46 to 12.13) | 5  (3 to 7) | 0.34  (0.19 to 0.5) | 121  (68 to 181) | 6.55  (3.76 to 9.7) | 0.31%  (0.08 to 0.53) | -0.06%  (-0.29 to 0.18) |
| Pakistan | 138  (80 to 200) | 0.28  (0.16 to 0.4) | 3228  (1868 to 4658) | 5.87  (3.42 to 8.43) | 365  (210 to 546) | 0.36  (0.21 to 0.53) | 8932  (5061 to 13468) | 7.53  (4.33 to 11.28) | 0.61%  (0.41 to 0.82) | 0.54%  (0.32 to 0.75) |
| Palau | 0  (0 to 0) | 1.15  (0.63 to 1.85) | 2  (1 to 4) | 25.34  (14.08 to 40.52) | 0  (0 to 0) | 1  (0.49 to 1.66) | 4  (2 to 7) | 20.21  (10.76 to 32.19) | -0.32%  (-0.41 to -0.24) | -0.65%  (-0.72 to -0.57) |
| Palestine | 13  (7 to 20) | 1.66  (0.97 to 2.54) | 307  (170 to 478) | 35.56  (20.09 to 55.21) | 29  (16 to 42) | 1.4  (0.79 to 2.03) | 711  (397 to 1044) | 28.29  (15.98 to 41.48) | -0.45%  (-0.63 to -0.26) | -0.64%  (-0.78 to -0.49) |
| Panama | 5  (3 to 7) | 0.36  (0.19 to 0.54) | 98  (54 to 148) | 6.8  (3.68 to 10.09) | 22  (11 to 33) | 0.48  (0.25 to 0.74) | 414  (218 to 629) | 9.34  (4.91 to 14.22) | 1.12%  (1.02 to 1.22) | 1.22%  (1.13 to 1.31) |
| Papua New Guinea | 4  (2 to 6) | 0.22  (0.12 to 0.36) | 117  (61 to 198) | 5.66  (2.99 to 9.38) | 9  (5 to 15) | 0.2  (0.11 to 0.31) | 293  (157 to 471) | 4.94  (2.7 to 7.87) | -0.49%  (-0.57 to -0.42) | -0.59%  (-0.68 to -0.51) |
| Paraguay | 5  (3 to 8) | 0.26  (0.13 to 0.42) | 98  (52 to 159) | 4.67  (2.44 to 7.52) | 23  (12 to 37) | 0.44  (0.21 to 0.72) | 444  (223 to 709) | 7.97  (4.02 to 12.92) | 2.08%  (1.94 to 2.22) | 2.06%  (1.93 to 2.18) |
| Peru | 45  (25 to 70) | 0.43  (0.23 to 0.66) | 848  (477 to 1314) | 7.55  (4.24 to 11.65) | 161  (86 to 262) | 0.49  (0.26 to 0.8) | 2875  (1569 to 4631) | 8.73  (4.76 to 14.08) | 0.34%  (0.16 to 0.53) | 0.35%  (0.16 to 0.53) |
| Philippines | 79  (44 to 123) | 0.35  (0.18 to 0.57) | 1806  (1022 to 2819) | 6.61  (3.68 to 10.29) | 311  (175 to 472) | 0.45  (0.25 to 0.69) | 7176  (4210 to 11013) | 9.04  (5.16 to 13.77) | 1.11%  (1.02 to 1.2) | 1.29%  (1.2 to 1.37) |
| Poland | 624  (366 to 879) | 1.47  (0.86 to 2.07) | 12077  (7156 to 17012) | 27.63  (16.38 to 39.13) | 1412  (841 to 2071) | 1.81  (1.08 to 2.63) | 24055  (14366 to 34083) | 32.03  (19.27 to 45.02) | 0.37%  (0.21 to 0.54) | 0.27%  (0.13 to 0.41) |
| Portugal | 259  (157 to 374) | 1.99  (1.21 to 2.84) | 4609  (2830 to 6603) | 33.66  (20.82 to 47.69) | 440  (248 to 651) | 1.46  (0.83 to 2.14) | 6879  (4078 to 10072) | 25.99  (15.51 to 37.69) | -0.94%  (-1.09 to -0.79) | -0.75%  (-0.92 to -0.58) |
| Puerto Rico | 35  (20 to 50) | 1.02  (0.59 to 1.46) | 641  (375 to 896) | 17.99  (10.63 to 25.13) | 67  (38 to 102) | 0.8  (0.47 to 1.2) | 1164  (696 to 1739) | 15.94  (9.41 to 23.92) | -1.01%  (-1.3 to -0.71) | -0.59%  (-0.85 to -0.32) |
| Qatar | 1  (1 to 2) | 1.57  (0.92 to 2.28) | 33  (19 to 48) | 30.57  (17.91 to 44) | 7  (4 to 10) | 1.24  (0.7 to 1.91) | 201  (112 to 317) | 23.4  (13.18 to 35.93) | -0.55%  (-1.23 to 0.13) | -0.6%  (-1.21 to 0.01) |
| Republic of Korea | 223  (130 to 331) | 0.97  (0.57 to 1.45) | 5377  (3211 to 8034) | 19.04  (11.23 to 28.17) | 891  (477 to 1364) | 0.96  (0.51 to 1.47) | 15821  (8954 to 23842) | 16.97  (9.47 to 25.61) | -0.21%  (-0.37 to -0.05) | -0.54%  (-0.72 to -0.36) |
| Republic of Moldova | 33  (18 to 49) | 0.85  (0.48 to 1.26) | 701  (406 to 1062) | 16.54  (9.45 to 24.5) | 51  (28 to 76) | 0.83  (0.47 to 1.25) | 1021  (557 to 1569) | 16.77  (9.17 to 25.4) | 0.42%  (-0.11 to 0.96) | 0.57%  (0.08 to 1.06) |
| Romania | 174  (104 to 258) | 0.67  (0.39 to 0.99) | 3872  (2320 to 5847) | 13.95  (8.44 to 20.85) | 490  (273 to 730) | 1.2  (0.67 to 1.79) | 9166  (5157 to 13528) | 23.84  (13.32 to 35.21) | 1.66%  (1.47 to 1.85) | 1.44%  (1.24 to 1.64) |
| Russian Federation | 1568  (903 to 2185) | 0.93  (0.54 to 1.29) | 31865  (18168 to 44835) | 17.86  (10.21 to 25.03) | 2874  (1657 to 4165) | 1.17  (0.67 to 1.69) | 50375  (29199 to 73154) | 20.46  (11.87 to 29.51) | 0.7%  (0.58 to 0.82) | 0.29%  (0.18 to 0.4) |
| Rwanda | 6  (3 to 10) | 0.27  (0.14 to 0.44) | 152  (78 to 262) | 5.64  (3.02 to 9.36) | 12  (6 to 22) | 0.26  (0.13 to 0.49) | 269  (132 to 481) | 4.8  (2.45 to 8.68) | -0.66%  (-0.9 to -0.41) | -1.2%  (-1.48 to -0.93) |
| Saint Kitts and Nevis | 0  (0 to 1) | 1.18  (0.7 to 1.74) | 8  (5 to 12) | 21.72  (13.08 to 31.78) | 1  (0 to 1) | 1.21  (0.67 to 1.8) | 13  (7 to 20) | 21.25  (11.81 to 31.68) | 0.7%  (0.47 to 0.94) | 0.51%  (0.32 to 0.7) |
| Saint Lucia | 1  (0 to 1) | 0.88  (0.46 to 1.32) | 13  (7 to 19) | 15.25  (8.48 to 22.39) | 2  (1 to 3) | 0.75  (0.42 to 1.18) | 31  (17 to 50) | 13.13  (7.34 to 20.99) | -1.19%  (-1.47 to -0.92) | -1%  (-1.24 to -0.76) |
| Saint Vincent and the Grenadines | 1  (0 to 1) | 0.83  (0.45 to 1.19) | 10  (5 to 15) | 14.78  (7.82 to 21.11) | 1  (1 to 2) | 0.87  (0.46 to 1.32) | 22  (12 to 32) | 15.65  (8.6 to 22.89) | 0.23%  (0.05 to 0.41) | 0.2%  (0.05 to 0.35) |
| Samoa | 1  (0 to 1) | 0.89  (0.5 to 1.33) | 18  (10 to 26) | 20.57  (11.57 to 30.18) | 1  (1 to 2) | 0.9  (0.5 to 1.32) | 32  (18 to 47) | 21.47  (12 to 31.8) | 0.01%  (-0.05 to 0.07) | 0.12%  (0.06 to 0.18) |
| San Marino | 1  (0 to 1) | 1.83  (0.96 to 2.75) | 11  (6 to 17) | 30.98  (17.29 to 45.8) | 1  (0 to 1) | 0.87  (0.46 to 1.39) | 13  (7 to 20) | 15.57  (7.99 to 25.15) | -1.44%  (-1.8 to -1.09) | -1.29%  (-1.62 to -0.95) |
| Sao Tome and Principe | 0  (0 to 0) | 0.19  (0.09 to 0.32) | 2  (1 to 3) | 3.21  (1.76 to 5.27) | 0  (0 to 0) | 0.25  (0.12 to 0.42) | 4  (2 to 7) | 4.14  (2.14 to 6.65) | 1.1%  (1.04 to 1.16) | 0.91%  (0.86 to 0.97) |
| Saudi Arabia | 26  (15 to 42) | 0.55  (0.31 to 0.87) | 649  (368 to 1022) | 11.28  (6.51 to 17.57) | 112  (65 to 164) | 0.76  (0.46 to 1.12) | 3368  (1911 to 4967) | 16.24  (9.63 to 23.93) | 1.28%  (0.93 to 1.63) | 1.51%  (1.17 to 1.84) |
| Senegal | 8  (4 to 12) | 0.28  (0.15 to 0.43) | 178  (98 to 276) | 5.74  (3.16 to 8.84) | 24  (13 to 38) | 0.37  (0.21 to 0.6) | 525  (293 to 839) | 7.2  (3.98 to 11.5) | 1.08%  (0.95 to 1.21) | 0.9%  (0.75 to 1.05) |
| Serbia | 123  (68 to 191) | 1.43  (0.8 to 2.16) | 2437  (1330 to 3781) | 25.02  (13.83 to 38.17) | 210  (115 to 322) | 1.18  (0.64 to 1.82) | 3768  (2098 to 5655) | 21.74  (12.07 to 32.53) | -0.75%  (-0.85 to -0.64) | -0.58%  (-0.67 to -0.48) |
| Seychelles | 1  (0 to 1) | 0.98  (0.57 to 1.44) | 10  (6 to 14) | 17.01  (9.95 to 24.77) | 1  (1 to 2) | 1.28  (0.7 to 1.92) | 23  (12 to 34) | 21.43  (11.55 to 31.4) | 1.05%  (0.81 to 1.3) | 0.9%  (0.66 to 1.15) |
| Sierra Leone | 3  (2 to 5) | 0.19  (0.1 to 0.31) | 71  (38 to 114) | 3.65  (1.98 to 5.85) | 8  (4 to 12) | 0.25  (0.13 to 0.4) | 164  (90 to 267) | 4.69  (2.57 to 7.67) | 1.24%  (1.1 to 1.38) | 1.18%  (1.04 to 1.32) |
| Singapore | 25  (15 to 37) | 1.25  (0.73 to 1.93) | 595  (340 to 890) | 26.91  (15.92 to 40.64) | 60  (33 to 91) | 0.72  (0.39 to 1.11) | 1239  (703 to 1827) | 14.43  (8.24 to 21.29) | -1.97%  (-2.17 to -1.78) | -2.15%  (-2.36 to -1.95) |
| Slovakia | 71  (39 to 105) | 1.19  (0.65 to 1.76) | 1347  (735 to 2013) | 22.19  (12.05 to 33.13) | 120  (66 to 190) | 1.23  (0.67 to 1.96) | 2142  (1192 to 3247) | 21.95  (12.14 to 33.4) | 0.13%  (0.04 to 0.21) | -0.05%  (-0.11 to 0.02) |
| Slovenia | 30  (17 to 45) | 1.19  (0.68 to 1.78) | 534  (306 to 815) | 21.32  (12.18 to 32.02) | 51  (27 to 79) | 0.97  (0.52 to 1.48) | 771  (411 to 1156) | 15.73  (8.4 to 23.59) | -0.8%  (-1.08 to -0.52) | -1.16%  (-1.43 to -0.9) |
| Solomon Islands | 1  (0 to 1) | 0.64  (0.35 to 1.02) | 22  (11 to 36) | 15.08  (7.93 to 24.97) | 2  (1 to 3) | 0.68  (0.36 to 1.07) | 60  (33 to 97) | 16.12  (8.62 to 25.71) | 0.21%  (0.08 to 0.33) | 0.23%  (0.08 to 0.38) |
| Somalia | 4  (2 to 8) | 0.24  (0.12 to 0.42) | 115  (52 to 213) | 5.1  (2.52 to 9.2) | 11  (5 to 19) | 0.26  (0.12 to 0.47) | 289  (134 to 531) | 5.27  (2.53 to 9.2) | 0.47%  (0.41 to 0.52) | 0.22%  (0.17 to 0.26) |
| South Africa | 168  (98 to 254) | 0.93  (0.54 to 1.41) | 3786  (2178 to 5643) | 18.46  (10.71 to 27.58) | 467  (290 to 640) | 1.18  (0.72 to 1.61) | 10489  (6383 to 14484) | 23.26  (14.29 to 31.84) | 0.66%  (0.44 to 0.89) | 0.73%  (0.53 to 0.93) |
| South Sudan | 5  (2 to 8) | 0.22  (0.11 to 0.38) | 108  (50 to 186) | 4.44  (2.13 to 7.58) | 8  (4 to 13) | 0.28  (0.13 to 0.47) | 193  (95 to 339) | 5.44  (2.63 to 9.06) | 0.84%  (0.74 to 0.95) | 0.73%  (0.6 to 0.86) |
| Spain | 872  (514 to 1285) | 1.58  (0.93 to 2.34) | 15370  (9403 to 22080) | 27.69  (16.96 to 39.89) | 1619  (906 to 2379) | 1.33  (0.75 to 1.96) | 25255  (14470 to 37055) | 23.7  (13.64 to 34.54) | -0.46%  (-0.59 to -0.33) | -0.43%  (-0.53 to -0.32) |
| Sri Lanka | 10  (5 to 16) | 0.12  (0.06 to 0.19) | 230  (116 to 364) | 2.27  (1.17 to 3.54) | 30  (15 to 51) | 0.12  (0.06 to 0.2) | 652  (334 to 1144) | 2.41  (1.24 to 4.14) | 0.56%  (0.39 to 0.73) | 0.64%  (0.48 to 0.81) |
| Sudan | 88  (50 to 141) | 0.97  (0.56 to 1.53) | 2586  (1390 to 4268) | 25.53  (14.04 to 41.32) | 202  (108 to 350) | 1.05  (0.57 to 1.79) | 6060  (3219 to 10382) | 26.71  (14.4 to 46.1) | 0.32%  (0.26 to 0.38) | 0.23%  (0.18 to 0.28) |
| Suriname | 2  (1 to 3) | 0.89  (0.53 to 1.35) | 48  (28 to 70) | 18.82  (11.12 to 27.92) | 6  (4 to 9) | 1  (0.59 to 1.51) | 135  (81 to 204) | 21.35  (12.63 to 32.13) | 0.8%  (0.59 to 1) | 0.77%  (0.57 to 0.97) |
| Sweden | 196  (113 to 291) | 1.16  (0.67 to 1.68) | 3291  (1958 to 4799) | 20.69  (12.38 to 29.64) | 246  (140 to 366) | 0.94  (0.55 to 1.4) | 3802  (2262 to 5615) | 16.29  (9.62 to 24.18) | -0.65%  (-0.78 to -0.51) | -0.63%  (-0.74 to -0.51) |
| Switzerland | 127  (73 to 184) | 1.1  (0.64 to 1.6) | 2176  (1249 to 3129) | 19.96  (11.25 to 28.61) | 170  (92 to 257) | 0.77  (0.43 to 1.15) | 2667  (1502 to 3906) | 13.54  (7.63 to 19.78) | -1.22%  (-1.38 to -1.06) | -1.36%  (-1.52 to -1.2) |
| Syrian Arab Republic | 26  (15 to 39) | 0.57  (0.34 to 0.83) | 657  (378 to 974) | 12.32  (7.17 to 18.64) | 64  (35 to 101) | 0.59  (0.33 to 0.94) | 1539  (846 to 2483) | 11.99  (6.64 to 19.24) | 0.03%  (-0.11 to 0.17) | -0.21%  (-0.38 to -0.04) |
| Taiwan (Province of China) | 164  (95 to 239) | 1.15  (0.69 to 1.71) | 4301  (2512 to 6184) | 26.38  (15.52 to 38.18) | 691  (406 to 1043) | 1.6  (0.94 to 2.39) | 13974  (8257 to 20183) | 33.44  (19.92 to 48.36) | 0.7%  (0.36 to 1.04) | 0.43%  (0.15 to 0.71) |
| Tajikistan | 8  (4 to 12) | 0.31  (0.18 to 0.48) | 160  (88 to 244) | 6.15  (3.4 to 9.47) | 9  (5 to 14) | 0.2  (0.11 to 0.33) | 193  (106 to 308) | 3.84  (2.12 to 6.06) | -1.33%  (-1.58 to -1.08) | -1.46%  (-1.69 to -1.24) |
| Thailand | 200  (118 to 303) | 0.75  (0.44 to 1.12) | 4033  (2320 to 6120) | 12.97  (7.6 to 19.71) | 877  (477 to 1412) | 0.81  (0.44 to 1.3) | 15641  (8662 to 25448) | 14.42  (7.92 to 23.41) | -0.06%  (-0.2 to 0.08) | 0.04%  (-0.09 to 0.17) |
| Timor-Leste | 0  (0 to 1) | 0.21  (0.11 to 0.36) | 11  (5 to 20) | 4.26  (2.11 to 7.15) | 2  (1 to 3) | 0.22  (0.11 to 0.38) | 35  (17 to 59) | 4.29  (2.14 to 7.18) | 0.21%  (0.04 to 0.38) | 0.03%  (-0.14 to 0.2) |
| Togo | 1  (1 to 2) | 0.13  (0.06 to 0.22) | 25  (13 to 41) | 2.34  (1.21 to 3.81) | 5  (2 to 8) | 0.19  (0.09 to 0.32) | 100  (49 to 167) | 3.25  (1.61 to 5.43) | 1.45%  (1.36 to 1.53) | 1.21%  (1.14 to 1.28) |
| Tokelau | 0  (0 to 0) | 0.65  (0.36 to 1.05) | 0  (0 to 0) | 15.15  (8.6 to 24.31) | 0  (0 to 0) | 0.57  (0.31 to 0.91) | 0  (0 to 0) | 13.04  (7.42 to 20.29) | -0.44%  (-0.46 to -0.43) | -0.54%  (-0.56 to -0.51) |
| Tonga | 0  (0 to 0) | 0.51  (0.29 to 0.77) | 7  (4 to 10) | 11.66  (6.68 to 17.27) | 0  (0 to 1) | 0.52  (0.3 to 0.8) | 9  (5 to 14) | 11.28  (6.49 to 17.25) | 0.11%  (0.02 to 0.2) | -0.13%  (-0.21 to -0.06) |
| Trinidad and Tobago | 11  (6 to 15) | 1.43  (0.83 to 2) | 235  (143 to 334) | 28.99  (17.55 to 40.99) | 26  (15 to 39) | 1.33  (0.77 to 2.02) | 589  (342 to 902) | 29.96  (17.67 to 45.68) | -0.13%  (-0.26 to -0.01) | 0.14%  (0.02 to 0.25) |
| Tunisia | 21  (12 to 33) | 0.56  (0.32 to 0.86) | 417  (235 to 640) | 9.55  (5.5 to 14.62) | 63  (35 to 100) | 0.54  (0.3 to 0.86) | 1177  (645 to 1882) | 9.36  (5.1 to 14.72) | -0.3%  (-0.38 to -0.22) | -0.18%  (-0.24 to -0.12) |
| Turkmenistan | 4  (2 to 6) | 0.26  (0.15 to 0.39) | 95  (55 to 144) | 5.28  (3.03 to 7.76) | 7  (4 to 11) | 0.23  (0.12 to 0.35) | 165  (86 to 257) | 4.48  (2.33 to 7.03) | -0.51%  (-1.05 to 0.03) | -0.54%  (-1.07 to -0.01) |
| Tuvalu | 0  (0 to 0) | 0.71  (0.41 to 1.07) | 1  (1 to 2) | 16.66  (9.61 to 25.15) | 0  (0 to 0) | 0.63  (0.34 to 0.98) | 2  (1 to 2) | 14.33  (7.73 to 21.51) | -0.42%  (-0.45 to -0.39) | -0.55%  (-0.58 to -0.51) |
| Uganda | 13  (7 to 22) | 0.25  (0.13 to 0.42) | 293  (155 to 489) | 4.91  (2.6 to 8.22) | 32  (17 to 54) | 0.29  (0.15 to 0.49) | 728  (388 to 1234) | 5.41  (2.9 to 9.03) | -0.05%  (-0.28 to 0.18) | -0.22%  (-0.48 to 0.04) |
| Ukraine | 516  (285 to 776) | 0.73  (0.4 to 1.07) | 10462  (5896 to 15377) | 14.34  (8.04 to 21.61) | 469  (253 to 755) | 0.57  (0.31 to 0.9) | 9164  (4918 to 14799) | 11.35  (5.93 to 18.38) | -0.88%  (-1.03 to -0.72) | -0.84%  (-0.97 to -0.71) |
| United Arab Emirates | 5  (3 to 9) | 1.56  (0.81 to 2.56) | 158  (78 to 255) | 34.51  (17.9 to 56.6) | 24  (13 to 40) | 1.27  (0.69 to 2.08) | 746  (401 to 1276) | 23.82  (12.96 to 38.63) | 1.32%  (0.71 to 1.94) | 0.42%  (-0.08 to 0.93) |
| United Kingdom | 3238  (1996 to 4414) | 3.37  (2.09 to 4.62) | 59991  (36736 to 81730) | 65.44  (39.47 to 88.95) | 3318  (2060 to 4535) | 2.24  (1.4 to 3.06) | 55706  (34820 to 75660) | 41.69  (25.65 to 56.66) | -1.32%  (-1.42 to -1.21) | -1.48%  (-1.58 to -1.39) |
| United Republic of Tanzania | 7  (4 to 13) | 0.09  (0.04 to 0.16) | 173  (86 to 294) | 1.7  (0.85 to 2.96) | 18  (9 to 32) | 0.09  (0.04 to 0.17) | 395  (207 to 675) | 1.71  (0.84 to 2.89) | 0.11%  (0.04 to 0.18) | -0.19%  (-0.28 to -0.1) |
| United States of America | 4342  (2476 to 6546) | 1.27  (0.73 to 1.92) | 75310  (43545 to 109529) | 22.63  (13.04 to 32.99) | 5135  (3039 to 7306) | 0.82  (0.48 to 1.17) | 90582  (53361 to 129752) | 15.26  (8.9 to 21.79) | -1.62%  (-1.74 to -1.5) | -1.42%  (-1.61 to -1.23) |
| United States Virgin Islands | 1  (1 to 1) | 1.43  (0.8 to 2.15) | 17  (10 to 25) | 23.54  (13.58 to 35.2) | 2  (1 to 2) | 0.84  (0.44 to 1.32) | 25  (14 to 38) | 13.47  (7.18 to 20.66) | -1.53%  (-1.83 to -1.23) | -1.68%  (-1.93 to -1.42) |
| Uruguay | 51  (28 to 78) | 1.29  (0.7 to 1.97) | 990  (568 to 1471) | 25.17  (14.4 to 37.53) | 87  (47 to 138) | 1.39  (0.75 to 2.19) | 1512  (829 to 2368) | 26.81  (14.73 to 41.31) | 0.13%  (0.05 to 0.22) | 0.14%  (0.06 to 0.21) |
| Uzbekistan | 16  (9 to 24) | 0.15  (0.09 to 0.23) | 341  (199 to 529) | 3.06  (1.77 to 4.78) | 25  (14 to 40) | 0.11  (0.06 to 0.18) | 597  (329 to 953) | 2.34  (1.28 to 3.78) | -0.72%  (-1.13 to -0.32) | -0.8%  (-1.24 to -0.36) |
| Vanuatu | 0  (0 to 0) | 0.19  (0.1 to 0.35) | 2  (1 to 4) | 3.69  (1.87 to 6.57) | 0  (0 to 0) | 0.2  (0.09 to 0.36) | 5  (3 to 10) | 3.66  (1.73 to 6.3) | -0.06%  (-0.11 to -0.01) | -0.27%  (-0.38 to -0.16) |
| Venezuela (Bolivarian Republic of) | 32  (18 to 49) | 0.39  (0.21 to 0.59) | 682  (392 to 1028) | 7.37  (4.2 to 11.18) | 122  (66 to 194) | 0.44  (0.24 to 0.7) | 2550  (1354 to 3926) | 8.66  (4.7 to 13.51) | 0.5%  (0.41 to 0.59) | 0.56%  (0.47 to 0.64) |
| Viet Nam | 91  (47 to 151) | 0.24  (0.13 to 0.4) | 2103  (1063 to 3621) | 5.26  (2.69 to 8.84) | 368  (195 to 607) | 0.4  (0.21 to 0.66) | 8781  (4540 to 14680) | 8.76  (4.6 to 14.62) | 1.87%  (1.74 to 1.99) | 1.99%  (1.87 to 2.11) |
| Yemen | 23  (12 to 39) | 0.57  (0.32 to 0.93) | 616  (319 to 1052) | 12.56  (6.64 to 20.96) | 74  (38 to 119) | 0.61  (0.32 to 1.01) | 1912  (954 to 3095) | 13.29  (6.72 to 21.58) | 0.33%  (0.26 to 0.4) | 0.28%  (0.21 to 0.36) |
| Zambia | 7  (4 to 12) | 0.31  (0.17 to 0.49) | 186  (102 to 298) | 6.75  (3.71 to 10.46) | 23  (11 to 44) | 0.43  (0.2 to 0.77) | 581  (275 to 1119) | 8.82  (4.27 to 16.41) | 1.01%  (0.9 to 1.13) | 0.82%  (0.7 to 0.95) |
| Zimbabwe | 15  (8 to 23) | 0.5  (0.27 to 0.8) | 306  (170 to 475) | 8.8  (4.95 to 13.82) | 31  (18 to 49) | 0.63  (0.35 to 0.98) | 721  (404 to 1150) | 11.88  (6.77 to 18.3) | 1.12%  (0.74 to 1.52) | 1.39%  (0.96 to 1.82) |

**Supplementary Figure 1. Temporal trend of ASMR and ASDR of colon and rectal cancer attributable to low physical activity from 1990 to 2021 at global and SDI levels by sex.**


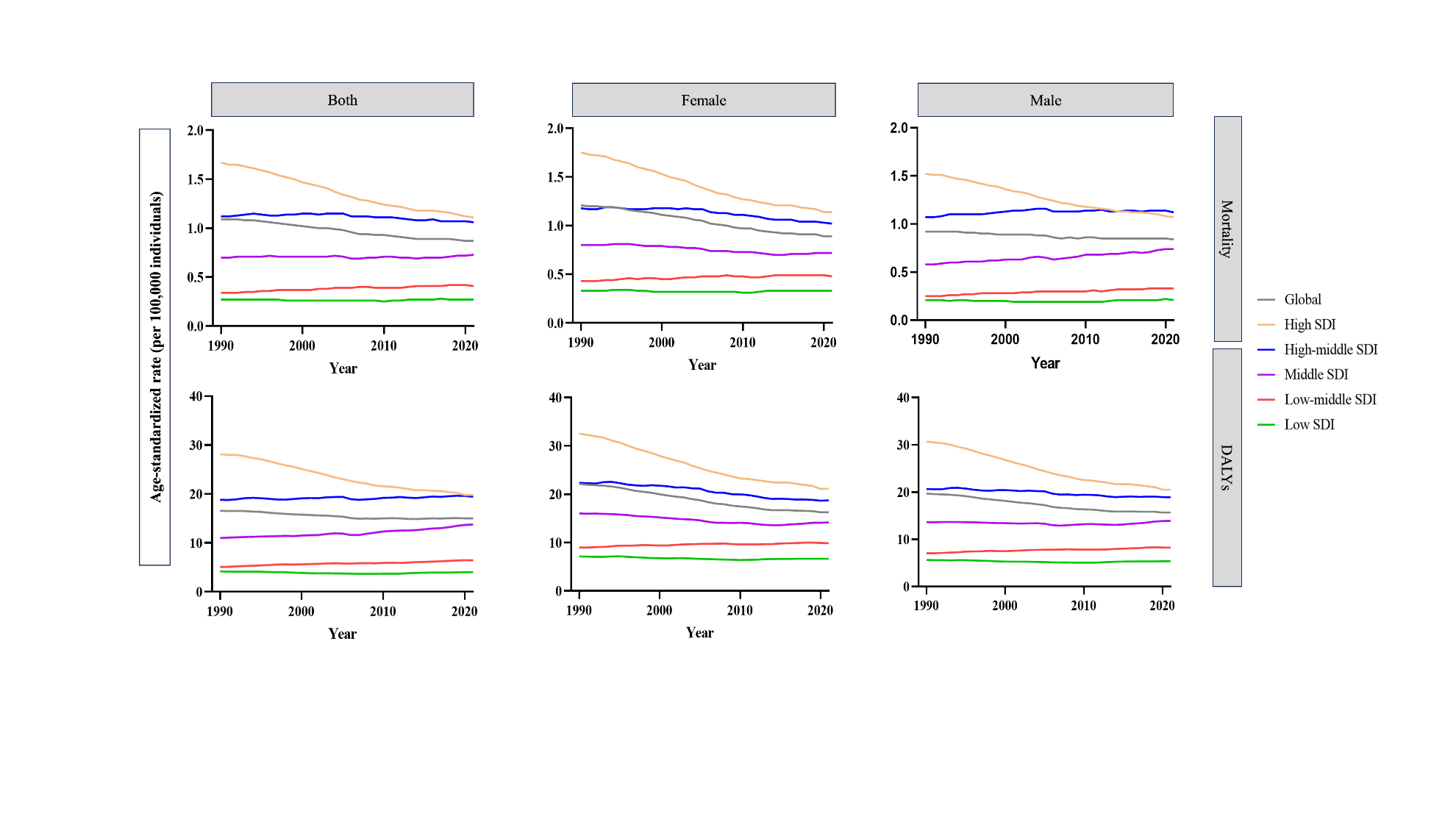


**Supplementary figure 2. EAPC of ASMR and ASDR of colon and rectal cancer attributable to low physical activity from 1990 to 2021 at SDI levels by sex.**


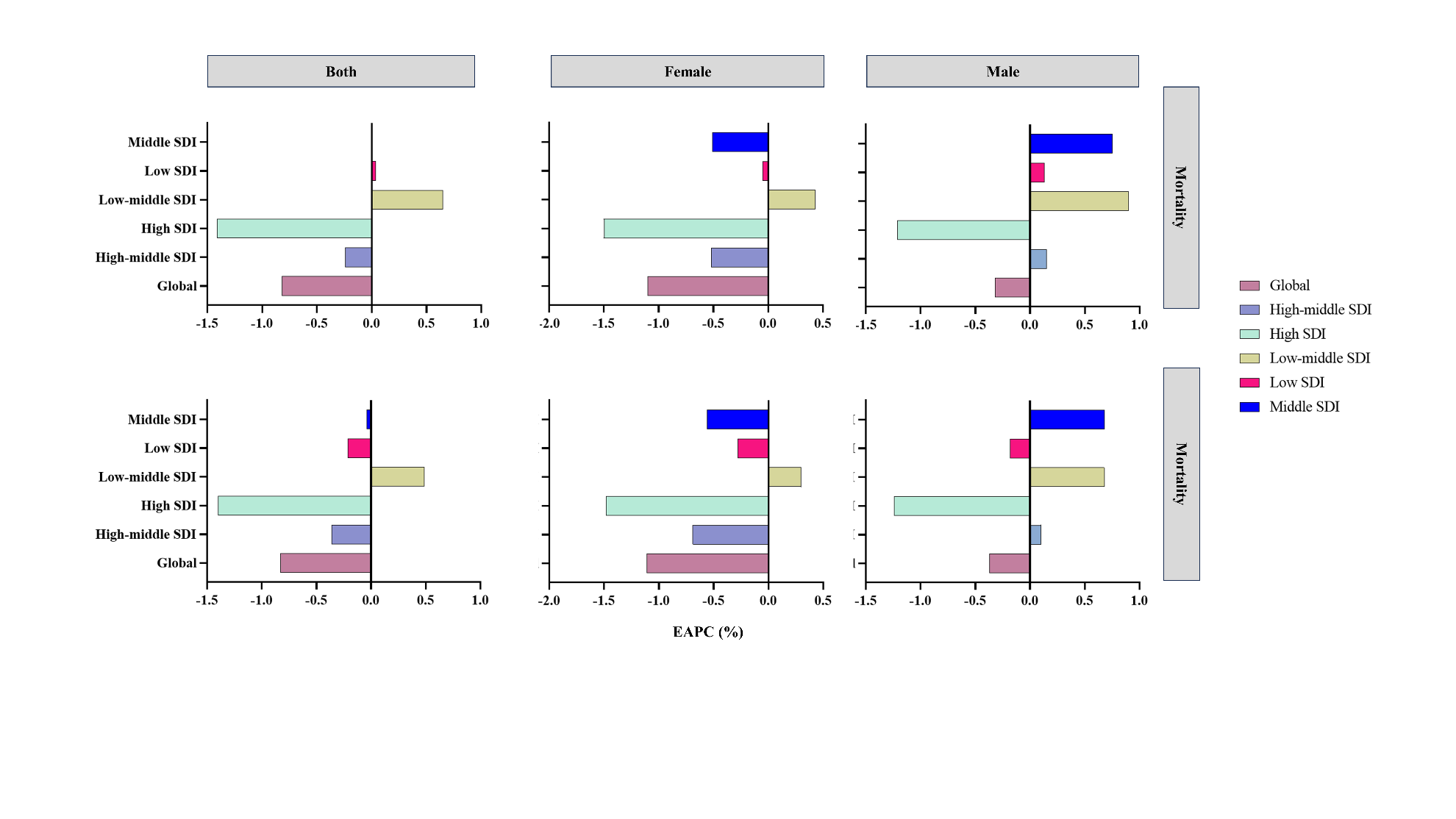


Supplementary figure 3. ASMR and ASDR of colon and rectal cancer attributable to low physical activity from 1990 to 2021 by age.


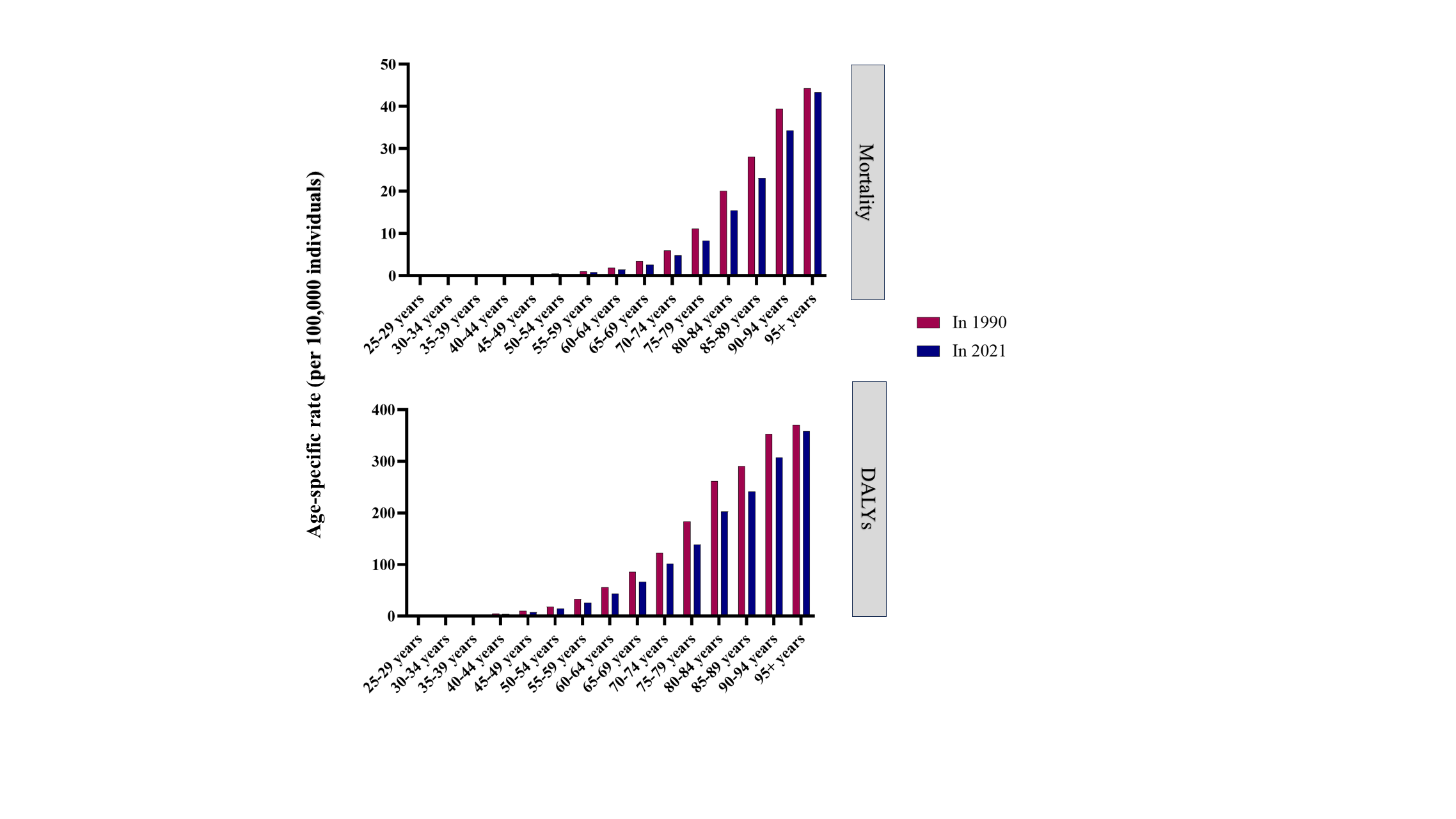


**Supplementary figure 4. EAPC of ASMR and ASDR of colon and rectal cancer attributable to low physical activity from 1990 to 2021 by sex and age.**


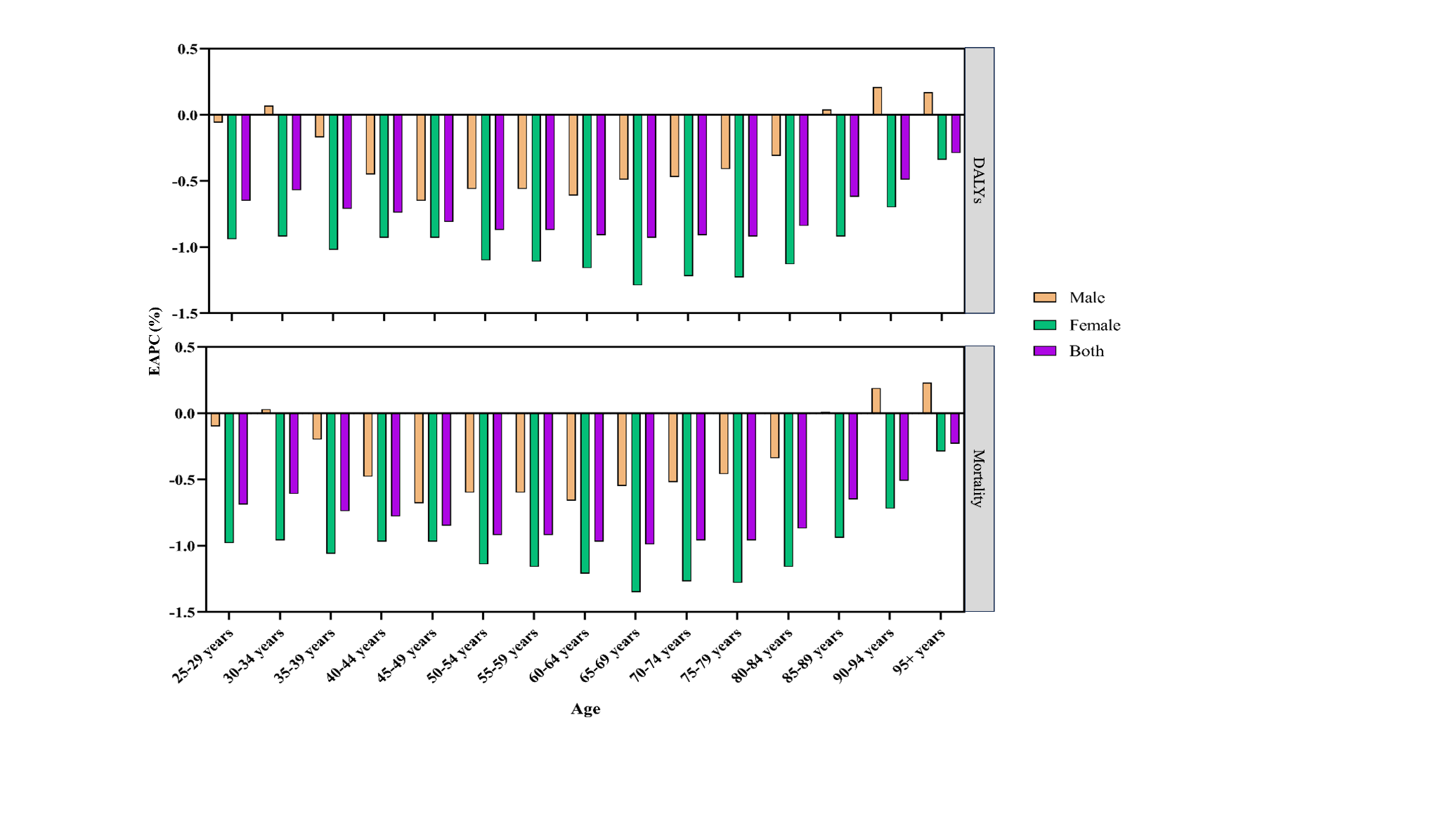


**Supplementary figure 5.** **EAPC of ASMR and ASDR of colon and rectal cancer attributable to low physical activity from 1990 to 2021 at regions levels by sex.**


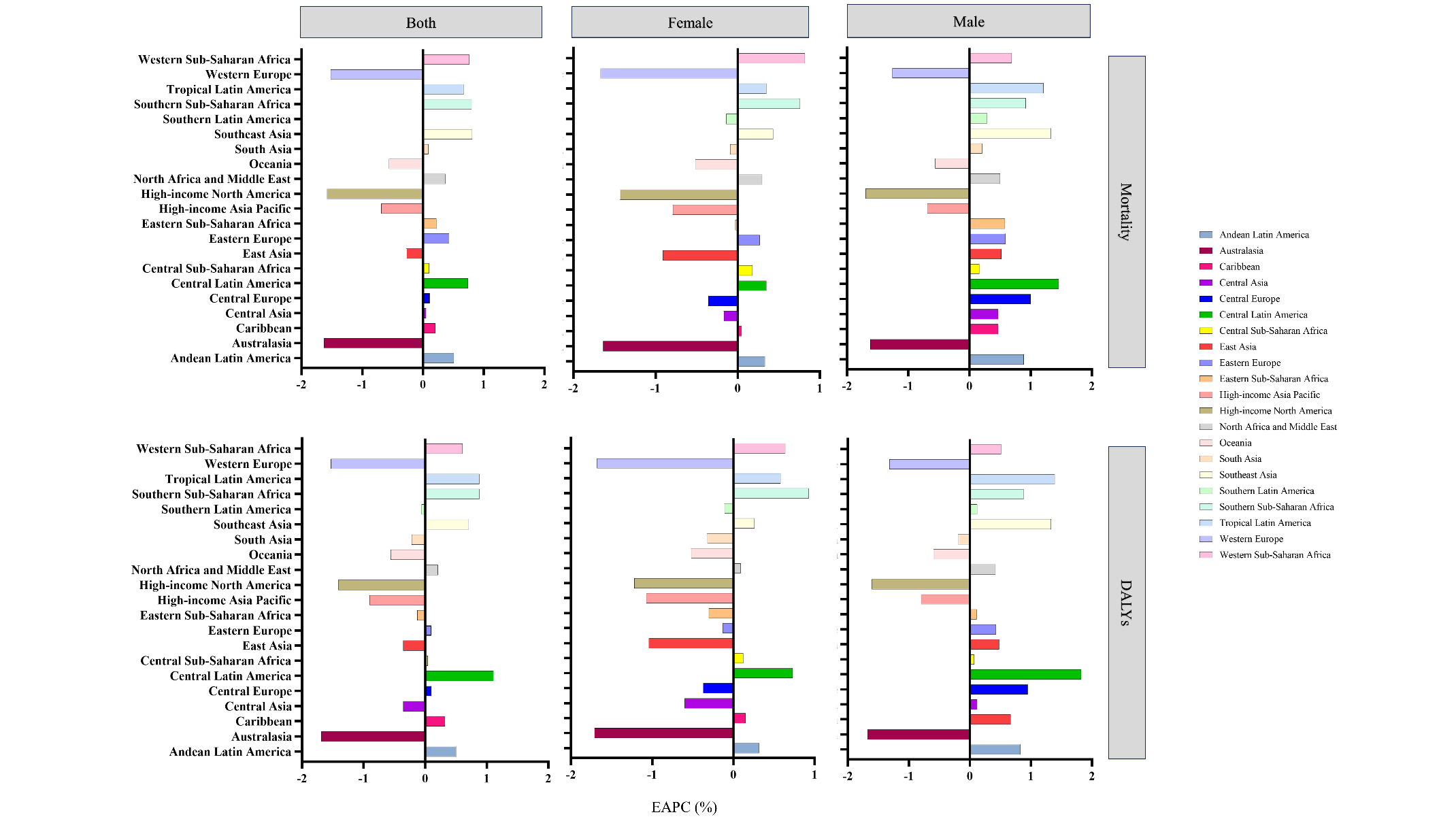

Supplement: Supplementary file 1 — Supplementary file1 (DOCX 1694 KB) [file 384_2025_4811_MOESM1_ESM.docx]
